# Enhanced efficacy of immune checkpoint inhibitors by folate-targeted multifunctional drug through synergistic therapy inducing ferroptosis and immunogenic cell death in bladder cancer

**DOI:** 10.1016/j.mtbio.2025.101584

**Published:** 2025-02-21

**Authors:** Yibo Shi, Guangrui Fan, Enguang Yang, Yuanfeng Zhang, Hui Ding, Junqiang Tian, Liang Cheng, Hanzhang Wang, Tianzhi Hao, Baodui Wang, Zhiping Wang

**Affiliations:** a Institute of Urology, The Second Hospital of Lanzhou University, Key Laboratory of Gansu Province for Urological Diseases, Gansu Province Clinical Research Center for Urinary System Disease, 730030, Lanzhou, Gansu, China; b The Legorreta Cancer Center at Brown University, Department of Pathology and Laboratory Medicine, The Warren Albert Medical School of Brown University, Brown University Health, Providence, RI, USA; c State Key Laboratory of Applied Organic Chemistry, Key Laboratory of Nonferrous Metal Chemistry and Resources Utilization of Gansu Province, Lanzhou University, 730000, Lanzhou, Gansu, China

**Keywords:** Composite multifunctional drug, Synergistic therapy, Bladder cancer, Immunogenic cell death, Immune checkpoint blockade

## Abstract

**Purpose:**

The research aims to elucidate the anti-tumor mechanism of the composite multifunctional folate-targeted drug DIFP-FA through sonodynamic therapy (SDT), chemodynamic therapy (CDT), and chemotherapy, as well as its potential to augment immune checkpoint blockade (ICB) therapy in bladder cancer (BC).

**Methods:**

DIFP-FA was synthesized *via* the W/O/W method. Its targeting efficacy was assessed using immunofluorescence and small animal imaging. Specific mechanisms were investigated through transcriptome sequencing and validation at both cellular and animal levels was conducted. BC patient-derived organoids (PDOs) and patient-derived tumor xenograft (PDX) models, derived from BC tissues resistant to cisplatin-gemcitabine and tislelizumab, were utilized to evaluate the efficacy of DIFP-FA in combination with SDT/CDT and chemotherapy. A humanized BC-PDX model was constructed to explore the synergistic effect of DIFP-FA with ICB therapy.

**Results:**

DIFP-FA, by incorporating doxorubicin and indocyanine green, leverages specific binding to folate receptors for precise targeting and efficient internalization into BC cells. DIFP-FA exhibits pH and ultrasound (US)-responsive cargo release properties, ensuring spatiotemporally controlled release. DIFP-FA induces reduced GPX4 and SLC7A11 expression and ferroptosis through the combination of SDT/CDT and chemotherapy. It also facilitates the transport and release of DAMPs, leading to immunogenic cell death (ICD). PDOs and PDX experiments demonstrated that DIFP-FA + US enhanced T lymphocyte infiltration in tumor tissues. Moreover, its combination with anti-PD-1 therapy effectively cleared immune-tolerant BC.

**Conclusions:**

DIFP-FA integrates SDT/CDT with chemotherapy to induce ferroptosis and ICD, efficiently eradicating tumors and activating the immune response, thereby enhancing the efficacy of ICB therapy.

## Highlights

• DIFP-FA achieves folate receptor-mediated cellular targeting and internalization, enhancing tumor-specific drug accumulation.
• pH and ultrasound-responsive release enables spatiotemporally controlled drug activation while protecting healthy tissues.
• DIFP-FA triggers ferroptosis/ICD *via* SDT/CDT/chemotherapy, boosting immune checkpoint inhibitors in bladder cancer.
• DIFP-FA synergizes with anti-PD-1 to drive T-cell infiltration, achieving complete ablation of drug-resistant bladder tumors.
• DIFP-FA-mediated SDT/CDT/chemotherapy synergizes with ICB, providing precise and effective therapy for immunotolerant tumors.

## Graphical abstract

Schematic illustration of the composite multifunctional targeted drug DIFP-FA inducing ferroptosis and immunogenic cell death (ICD) by integrating multiple treatment modalities, including chemotherapy, sonodynamic therapy (SDT), and chemodynamic therapy (CDT). Furthermore, when combined with anti-PD-1 therapy, it effectively eliminates immune-tolerant tumors.

**Figure:**
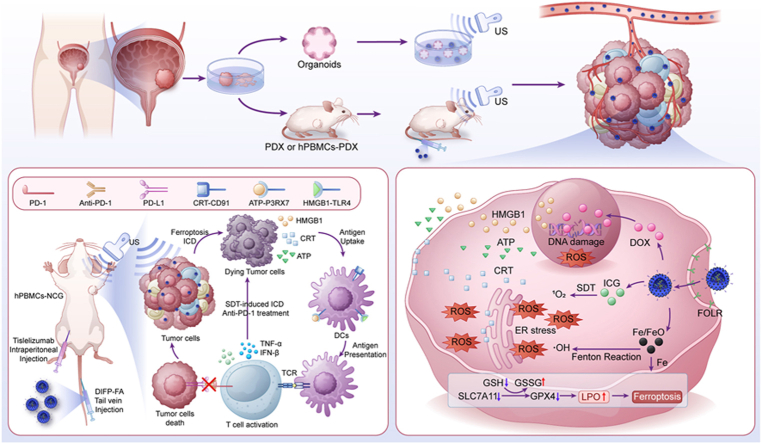

## 1 Introduction

Bladder cancer (BC) is a prevalent urological malignancy with significant morbidity and mortality rates [1]. Approximately 44 % of patients with advanced BC experience recurrence or disease progression despite conventional postoperative chemotherapy and transurethral resection of the bladder tumor (TURBT) [2]. Immune checkpoint blockade (ICB) therapy has emerged as a promising salvage treatment for patients with BC after the failure of first-line therapy [3]. However, its efficacy is compromised by tumor microenvironment (TME)-induced immune evasion and immunosuppression, resulting in a mere 20 % response rate in patients with BC [4,5]. Traditional monotherapy or single-modality treatments are inadequate for achieving precise and effective clinical outcomes. Thus, exploring novel drugs and therapeutic strategies tailored to BC characteristics is both critical and urgent.

The previously developed DOX-ICG@Fe/FeO-PPP-FA (DIFP-FA) integrates doxorubicin (DOX), indocyanine green (ICG), and Fe/FeO core-shell nanocrystals (NCs) [6]. DIFP-FA features a folic acid (FA)-modified Fe/FeO-PNIPAM-PEG-PLGA (PPP) shell encapsulating DOX and ICG. And DIFP-FA ensures controlled release in response to near-infrared (NIR) and TME, leveraging DOX chemotherapy and photodynamic therapy (PDT) for effective treatment [6]. However, NIR light's limited tissue penetration, less than 1 cm, renders it suboptimal for deep tumors like BC [7]. Conversely, ultrasound (US) can penetrate over 10 cm of tissue with minimal side effects [8], positioning sonodynamic therapy (SDT) as a superior clinical option. SDT offers precise, spatiotemporally controlled, and non-invasive tumor ablation. Additionally, ICG in DIFP-FA, beyond being a photosensitizer, is an effective sonosensitizer [9]. Nonetheless, it remains to be demonstrated whether DIFP-FA can accurately deliver DOX and ICG to BC cells and induce cytotoxicity *via* US. Furthermore, a comprehensive understanding of its mechanism of action is essential for clinical translation.

In this study, BC cells, patient-derived organoids (PDOs), and patient-derived tumor xenograft (PDX) models derived from BC tissues resistant to cisplatin-gemcitabine and recurrent after tislelizumab treatment were employed. They were used to assess the efficacy, safety, and mechanism of DIFP-FA in enhancing ICB therapy. DIFP-FA utilizes FA for targeted uptake into BC cells. The encapsulated chemotherapeutic agent DOX induces DNA damage, while ICG generates singlet oxygen (^1^O_2_) *via* SDT. Additionally, Fe/FeO NCs generate hydroxyl radicals (·OH) *via* the Fenton reaction for chemodynamic therapy (CDT). DIFP-FA achieves the efficient eradication of BC through the combination of sonodynamic, chemodynamic, and chemotherapy. Furthermore, the combination therapy of DIFP-FA induces ferroptosis and immunogenic cell death (ICD) in tumor cells. The resulting damage-associated molecular patterns (DAMPs) act as an in situ "vaccine" to stimulate the host's adaptive immune response. This occurs through the promotion of dendritic cells (DCs) maturation, cytokine secretion, and the infiltration of cytotoxic T lymphocytes. Notably, the combination therapy of DIFP-FA enhances the antitumor efficacy of PD-1 checkpoint blockade, leading to the complete eradication of resistant tumors *in vivo*.

## 2 Methods and materials

### 2.1 Preparation and characterization of DIFP-FA

Based on our previous research [6], DIFP-FA was synthesized *via* the W/O/W emulsion technique, encapsulating DOX and ICG within a Fe/FeO NCs and PPP shell (Fig. S1). Scanning electron microscopy (SEM) (Thermo Fisher, USA) provided images of DIFP-FA at 500 nm and 200 nm. Dynamic light scattering (DLS) and zeta potential measurements for PPP, Fe/FeO-PPP, DIFP, and DIFP-FA were conducted using a zeta potential and particle size analyzer (Brookhaven Instruments Corporation, USA) during the synthesis of DIFP-FA. The stability of DIFP-FA was assessed by incubating it in deionized water, saline, phosphate buffered saline (PBS), RPMI-1640, and RPMI-1640 with 10 % fetal bovine serum (FBS) respectively. DLS monitored the particle size of DIFP-FA after 24h of incubation. DIFP-FA was incubated in PBS at pH 7.4 and 5.4 for 40 h, with US irradiation applied for 1 min at the 4th hour. UV–Vis spectrophotometry detected the absorbance of the solutions at various time points. The DOX release rate was determined using a DOX concentration-absorbance standard curve.

### 2.2 Materials and instruments

The materials utilized in this study are detailed in Table S1. All US irradiation was performed using the LIPUSTIM® 330 Sonodynamic Therapy System (SXULTRASONIC, China) at 1.0 MHz frequency, 20 % duty cycle, and 500 mW/cm^2^. This study has been performed in accordance with the Declaration of Helsinki. All protocols and procedures were approved by the Medical Ethics Committee of the Lanzhou University Second Hospital (approval numbers: D2023-059 and 2023A-043). Written informed consent was obtained from each patient. All patient data were kept anonymous. Animal experiments adhered to the National Institutes of Health guidelines for the Care and Use of Laboratory Animals and the Regulations for the Administration of Affairs Concerning Experimental Animals (2017.03.01 edition) published by the State Council of the People's Republic of China. At the experiment's conclusion, animals were euthanized by an overdose of inhalation anesthetics.

### 2.3 Detection of ROS, ·OH, ^1^O_2_*in vitro*

Total reactive oxygen species (ROS) generation by US irradiation on DIFP-FA was evaluated using a methylene blue (MB) colorimetric indicator. The experiment comprised six groups: (1) Blank, (2) H_2_O_2_ + US, (3) DIFP-FA, (4) DIFP-FA + H_2_O_2_, (5) DIFP-FA + US, and (6) DIFP-FA + H_2_O_2_ + US. The concentration of DIFP-FA was 80 μg/mL, and the concentration of H_2_O_2_ was 10 mM. Groups H_2_O_2_ + US, DIFP-FA + US, and DIFP-FA + H_2_O_2_ + US were irradiated with US for 1 min. UV–Vis spectral changes were recorded for different MB subgroups.

^1^O_2_ generation by DIFP-FA *in vitro* was detected using 1,3-diphenylisobenzofuran (DPBF). 1 mL DIFP-FA (80 μg/mL) was added to 1 mL DPBF (0.02 mM) solution (pH 5.4). US was applied for 5 min, and the UV–Vis spectra of DPBF were recorded at the end of each minute.

·OH generation by DIFP-FA *in vitro* was demonstrated *via* terephthalic acid (TA) oxidation. Non-fluorescent TA converts to fluorescent hydroxyterephthalic acid (TAOH) upon oxidation by ·OH. 1 mL DIFP-FA (80 μg/mL) was added to 2 mL TA solution (1 mM) at pH 7.4, 6.5, and 5.4 respectively. The mixtures were irradiated with US for 5 min. The fluorescence spectra of TAOH were measured at the end of each minute (ex/em = 327 nm/420 nm). The fluorescence intensity of TAOH at 420 nm was used to calculate its fluorescence as a function of US irradiation time.

### 2.4 Detection of cellular uptake, cytotoxicity, intracellular ROS

Mouse MB49 BC cells and human UMUC-3, T24, HT-1376, 5637, RT4, and J82 BC cells were obtained from the Lanzhou University Second Hospital. BC cells were cultured in RPMI-1640 medium supplemented with 10 % FBS and 1 % penicillin/streptomycin at 37 °C under 5 % CO_2_. The expression level of folate receptor (FOLR) in BC cell lines MB49, HT-1376, 5637, T24, RT4, J82, and UMUC-3 was analyzed using western blotting (WB). UMUC-3 and T24, selected for their similar growth characteristics, were subjected to further experiments. Immunofluorescence was utilized to detect FOLR expression in these cell lines. UMUC-3 and T24 cells were seeded in confocal dishes and underwent the following treatments: (1) incubation with free ICG (5 μg/mL), DIFP (80 μg/mL), and DIFP-FA (80 μg/mL) for 4 h, and (2) incubation with DIFP-FA (80 μg/mL) for 0, 2, 4, and 6 h. The fluorescence intensity of ICG (ex/em = 780 nm/840 nm) was observed *via* a two-photon laser scanning confocal microscope (LSCM) (Carl Zeiss LSM880, Germany) to assess cellular uptake. Intracellular ICG fluorescence intensity was quantified using ImageJ.

UMUC-3 and T24 cells were incubated with varying concentrations of DIFP-FA (0, 10, 20, 40, 80, 160, 320 μg/mL) for 4 h, followed by 1 min of US irradiation. Cell viability was assessed using CCK8 assay kits and Calcein-AM and propidium iodide (AM/PI) assay kits according to the manufacturer's protocols. UMUC-3 and T24 cells were incubated with DIFP-FA (80 μg/mL) for 4 h, exposed to US for 1 min, and incubated for an additional 2 h. Intracellular ROS levels were detected using LSCM and flow cytometry assays with a ROS detection kit following the manufacturer's instructions.

### 2.5 Establishment of BC-PDOs and DIFP-FA-mediated killing

BC-PDOs were established as described by Lee et al. [10]. BC-PDOs were cultured in DMEM-F12 medium supplemented with 10 ng/mL epidermal growth factor (EGF), 5 % FBS, 10 μM Y-27632, and 1 % penicillin/streptomycin/amphotericin B at 37 °C under 5 % CO_2_. Fresh tumor tissues were transported on ice, washed, minced, and digested with collagenase/hyaluronidase. After centrifugation and resuspension, the tissues were treated with TrypLE Express, filtered through a 100 μm cell filter, mixed with Matrigel, and dispensed into 24-well plates at 20–30 μL per well. The droplets were incubated at 37 °C and 5 % CO_2_ for 30 min to solidify, followed by the addition of 1.5 mL organoid culture medium per well with medium replacement every 3 days. Upon establishing stable BC PDOs, the following treatments were administered: (1) US: US irradiation for 1 min; (2) DIFP-FA: 80 μg/mL DIFP-FA for 24 h; (3) DIFP-FA + US: 80 μg/mL DIFP-FA for 24 h followed by US irradiation for 1 min. According to the AM/PI assay kit protocol, the viability of PDOs was measured.

### 2.6 Transcriptome sequencing and analysis

UMUC-3 cells cultured in 6-well plates were treated with PBS or DIFP-FA + US (80 μg/mL DIFP-FA for 4 h followed by US irradiation for 1 min). Treated cells were harvested, quick-frozen in liquid nitrogen, and stored at −80 °C. Transcriptomic sequencing and analysis were performed by Frasergen Co. Ltd. in Wuhan, China.

### 2.7 Evaluation of cellular ferroptosis, GSH/GSSG ratio, LPO levels

UMUC-3 and T24 cells were seeded into 24-well plates (5 × 10^4^ cells per well) and assigned to five groups based on different intervention conditions: (1) Blank: PBS only; (2) US: US irradiation for 1 min; (3) DIFP-FA: 80 μg/mL DIFP-FA for 4 h; (4) DIFP-FA + US: 80 μg/mL DIFP-FA for 4 h followed by US irradiation for 1 min; (5) DIFP-FA + US + Fer-1: the same conditions as the DIFP-FA + US group, with the addition of 0.5 μM Ferrostatin-1 (Fer-1). Cell viability was evaluated using the CCK8 assay kit. Intracellular protein levels of SLC7A11/xCT, GPX4, and GAPDH were analyzed *via* WB. UMUC-3 and T24 cells from blank, US, DIFP-FA, and DIFP-FA + US groups were collected, and intracellular levels of reduced glutathione (GSH), oxidized glutathione (GSSG), and lipid peroxidation (LPO) were quantified using the GSH and GSSG Assay Kit and Lipid Peroxidation Malondialdehyde (MDA) Assay Kit, in accordance with the manufacturer's protocols.

### 2.8 Detection of intracellular translocation and release of DAMPs

In UMUC-3 and T24 cells from blank, US, DIFP-FA, and DIFP-FA + US groups (The treatment conditions are the same as in section 2.7), the expression of high mobility group box 1 (HMGB1) and calreticulin (CRT) was determined through immunofluorescence and WB, respectively. HMGB1 levels in cell supernatants were quantified using an enzyme-linked immunosorbent assay (ELISA) kit. Intracellular and extracellular adenosine triphosphate (ATP) levels were measured using the ATP assay kit.

### 2.9 Establishment of a BC-PDX model, evaluation of *in vivo* anti-tumor effect and biosafety

Male NCG (NOD/ShiLtJGpt-*Prkdc*^*em26Cd52*^*Il2rg*^*em26Cd22*^/Gpt) mice aged 4–5 weeks were procured from GemPharmatech Co. Ltd. (China). Tumor tissue from a patient with BC that was resistant to both cisplatin and gemcitabine, as well as recurrent after tislelizumab treatment, were used to establish a PDX model in NCG mice. The diagnosis and treatment of the patient and the timing of obtaining the resistant tumor tissue are shown in Fig. S2. Freshly excised bladder tumor tissues were placed in 1640 medium supplemented with 10 % FBS, 1 % penicillin/streptomycin/amphotericin B, and transported on ice to the laboratory. Tumor tissues were cut into 3 × 3 × 3 mm pieces, incubated in Matrigel for approximately 10 min, and then implanted into the axillary subcutis of mice as the 1st generation PDX (G1). When tumor graft volumes reached approximately 1000 mm^3^, they were excised and transplanted into new NCG mice for subsequent passaging as the 2nd generation PDX (G2). Using the same method, the PDX tumor was inoculated into the 3rd generation (G3) for further experiments.

When G3 BC-PDX tumors reached approximately 100 mm^3^, the mice were randomly assigned to four groups (n = 5 per group): (1) Saline: intravenous saline injection; (2) US: US irradiation; (3) DIFP-FA: intravenous injection of 20 mg/kg DIFP-FA; and (4) DIFP-FA + US: intravenous injection of 20 mg/kg DIFP-FA followed by US irradiation. Each injection consisted of 200 μL of saline or drug, with US irradiation conducted for 5 min on the 1st and 3rd days. The body weight and tumor size of the mice were recorded every two days, with tumor volume calculated using the formula: volume = length × width^2^/2. After 14 days, the mice were euthanized, and blood and tumor tissues were collected from each group. The weight of each tumor was recorded. Heart, liver, spleen, lung, kidney, intestine, and skin sections underwent hematoxylin-eosin (H&E) staining. Additionally, tumor specimens were subjected to immunohistochemistry (IHC) or immunofluorescent staining for GPX4, SLC7A11, CRT, HMGB1, and Ki67. Blood samples were analyzed for routine blood examination and biochemical tests, including red blood cell (RBC), hemoglobin (HGB), platelet (PLT), liver function markers (aspartate aminotransferase (AST), alanine aminotransferase (ALT)), renal function markers (blood urea nitrogen (BUN), creatinine (CREA)), and cardiac function markers (creatine kinase (CK), lactate dehydrogenase (LDH)).

### 2.10 Evaluation of immune activation of DIFP-FA synergistic therapy and antitumor effects of combination with PD-1

Approximately one week following the inoculation of G3 BC-PDX tumor tissue, NCG mice were injected with 5 × 10^6^ human peripheral blood mononuclear cells (hPBMCs) *via* the tail vein. Blood samples were collected for routine examination about one week later. The hPBMCs-NCG mice were then randomly divided into four groups (n = 5 per group): (1) Saline: intravenous saline injection; (2) PD-1: intraperitoneal injection of 10 mg/kg tislelizumab; (3) DIFP-FA + US: intravenous injection of 20 mg/kg DIFP-FA followed by US irradiation; and (4) DIFP-FA + US + PD-1: intravenous injection of 20 mg/kg DIFP-FA and intraperitoneal injection of 10 mg/kg tislelizumab followed by US irradiation. Each DIFP-FA injection was 200 μL, with US irradiation for 5 min on the 1st and 3rd days; tislelizumab was injected at 200 μL on the 2nd, 4th, and 7th days. After 21 days, the mice were euthanized, and blood and tumor tissues were collected. Tumor necrosis factor-α (TNF-α) and Interferon-γ (IFN-γ) levels in tumor tissues were quantified using TNF-α and IFN-γ ELISA kits respectively. Tumor tissues were stained with H&E and IHC for CD4 and CD8 to assess lymphocyte infiltration and immune activation, as well as the potential enhancement of ICB therapy in combination with PD-1.

### 2.11 Biodistribution of DIFP-FA *in vivo*

At 0, 12, 24, and 48 h post tail vein injection of DIFP-FA (20 mg/kg, 200 μL) in BC-PDX mice, the dynamic biodistribution of DIFP-FA was assessed using a DPM wide-spectrum small animal *in vivo* optical imaging system (DPM, China). At 24 and 48 h post-injection, the organ-specific biodistribution of DIFP-FA was evaluated by the DPM system after the mice were sacrificed, recording the fluorescence intensity in major organs (heart, liver, spleen, lung, kidney, and tumor).

### 2.12 Statistical analysis

All data were presented as mean ± standard deviation. Statistical analysis was performed using a two-tailed Student's t-test for two-group comparisons and a one-way analysis of variance for multiple groups. N.S. denotes no statistical significance, while *P* < 0.05 means statistical significance. Significance levels are denoted as ∗*P* < 0.05, ∗∗*P* < 0.01, ∗∗∗*P* < 0.001, and ∗∗∗∗*P* < 0.0001.

## 3 Results

### 3.1 Characterization and *in vitro* ROS generation of DIFP-FA

DIFP-FA exhibits a spherical structure with a uniform particle size distribution (Fig. 1A–B). DLS analysis revealed that the hydrodynamic diameter of DIFP-FA increased to 215.8 ± 59 nm after loading Fe/FeO NCs, DOX, ICG, and FA (Fig. 1C, Fig. S3). The polydispersity index (PDI) of DIFP-FA was 0.292 (Fig. S3D), and the zeta potential was −13.112 mV (Fig. 1D). Post-incubation in various solvents for 24 h, DIFP-FA maintained consistent hydrodynamic diameters and PDIs (Fig. 1E). The drug loading capacity of DIFP-FA was approximately 17.89 % (Fig. S4). As illustrated in Fig. 1F, US irradiation significantly enhanced the release of DOX from DIFP-FA after 4 h of incubation, confirming DIFP-FA's US-responsive cargo-releasing capability. Furthermore, after 40 h of treatment, the cumulative DOX release from DIFP-FA in an acidic environment was greater than in a neutral one (pH 5.4: 81.63 % vs pH 7.4: 47.93 %, *P* < 0.001), demonstrating DIFP-FA's pH-responsive DOX release pattern.

**Fig. 1:**
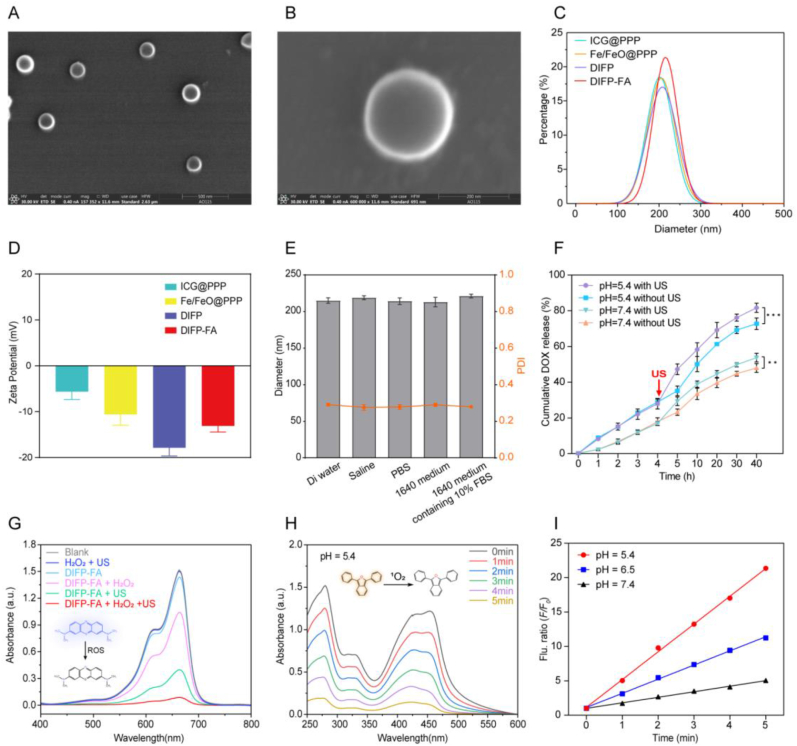
Characterization and ROS generation *in vitro* of DIFP-FA. SEM images of DIFP-FA at 500 nm (A) and 200 nm (B). Hydrodynamic diameters (C) and Zeta potentials (D) of nanoparticles at various synthesis stages. (E) Hydrodynamic dimensions and PDI of DIFP-FA after 24 h of incubation in deionized water, saline, PBS, 1640 medium, and 1640 medium containing 10 % FBS (Error bars, mean ± SD, n = 3). (F) DOX release rate from DIFP-FA over time after incubation in PBS with pH 5.5 or 7.4, with US irradiation applied at the end of the 4th hour (Error bars, mean ± SD, n = 3). (G) UV–vis absorption spectra of MB to detect ROS generation induced by DIFP-FA under various conditions. Insets: Blue MB oxidized to colorless by ROS. (H) UV–vis absorption spectra of DPBF to detect ^1^O_2_ generation induced by DIFP-FA under different US exposure times. Insets: Yellow DPBF oxidized to colorless by ^1^O_2_. (I) Fluorescence intensity of TAOH at 420 nm as a function of incubation time in DIFP-FA solutions at pH 5.4, 6.5, and 7.4. *F*_*0*_ and *F* represent fluorescence intensities of the system without or with treatment, respectively. (For interpretation of the references to color in this figure legend, the reader is referred to the Web version of this article.)

Compared with the "Blank" and "H_2_O_2_ + US" groups, the "DIFP-FA + H_2_O_2_″ and "DIFP-FA + US" groups showed reduced MB absorbance at 655 nm, with the most substantial reduction observed in the "DIFP-FA + H_2_O_2_ + US" group (Fig. 1G). This illustrates that DIFP-FA can efficiently generate ROS under US stimulation. Additionally, the types of ROS produced by DIFP-FA under SDT were evaluated. The characteristic DPBF peak (λ = 466 nm) gradually diminished with prolonged "DIFP-FA + US" treatment time, illustrating sustained ^1^O_2_ generation. After 5 min of US irradiation, the DPBF absorbance in the "DIFP-FA + H_2_O_2_ + US" group was almost entirely eliminated, showing the highest ^1^O_2_ production (Fig. 1H). TAs at pH 7.4 (Fig. S5A), 6.5 (Fig. S5B), and 5.4 (Fig. S5C) were incubated with DIFP-FA, and their fluorescence spectra were detected post-US stimulation. Results illustrated that with increasing US treatment time, the TAOH fluorescence signal intensified, implying increased ·OH production. At pH 5.4, DIFP-FA produced maximum fluorescence enhancement (Fig. S5), illustrating that DIFP-FA's ·OH generation capacity is strongest in an acidic environment (Fig. 1I).

### 3.2 Cellular uptake and *in vivo* ROS production of DIFP-FA

Immunofluorescence and WB revealed that BC cells, including UMUC-3 and T24, highly express FOLR (Fig. 2A, Fig. S6). DIFP-FA demonstrated significantly higher intracellular fluorescence intensity compared with DIFP and free ICG under identical incubation conditions (Fig. 2B). Quantitative analysis of the average fluorescence intensity showed a 1.5- to 2-fold increase in cellular uptake of DIFP-FA compared with DIFP (UMUC-3: 2.04-fold, T24: 1.45-fold; *P* < 0.0001) (Fig. S7). These findings illustrate that folate modification enhances the uptake of DIFP-FA by BC cells. Additionally, the accumulation of DIFP-FA within both UMUC-3 and T24 cells progressively increased over time (Fig. S8A). Quantitative analysis of the mean fluorescence intensity revealed a significant increase in DIFP-FA fluorescence in both UMUC-3 and T24 cells after 4 h of incubation (Figs. S8B–C). Although the average fluorescence intensity continued to rise after 6 h, no statistical difference was observed compared with the 4-h incubation period (UMUC-3, 6 h vs. 4 h: 30.21 vs. 29.04; T24, 6 h vs. 4 h: 22.2 vs. 20.98, *P* > 0.05) (Fig. S8). Thus, 4 h was selected as the optimal DIFP-FA incubation time for subsequent experiments.

**Fig. 2:**
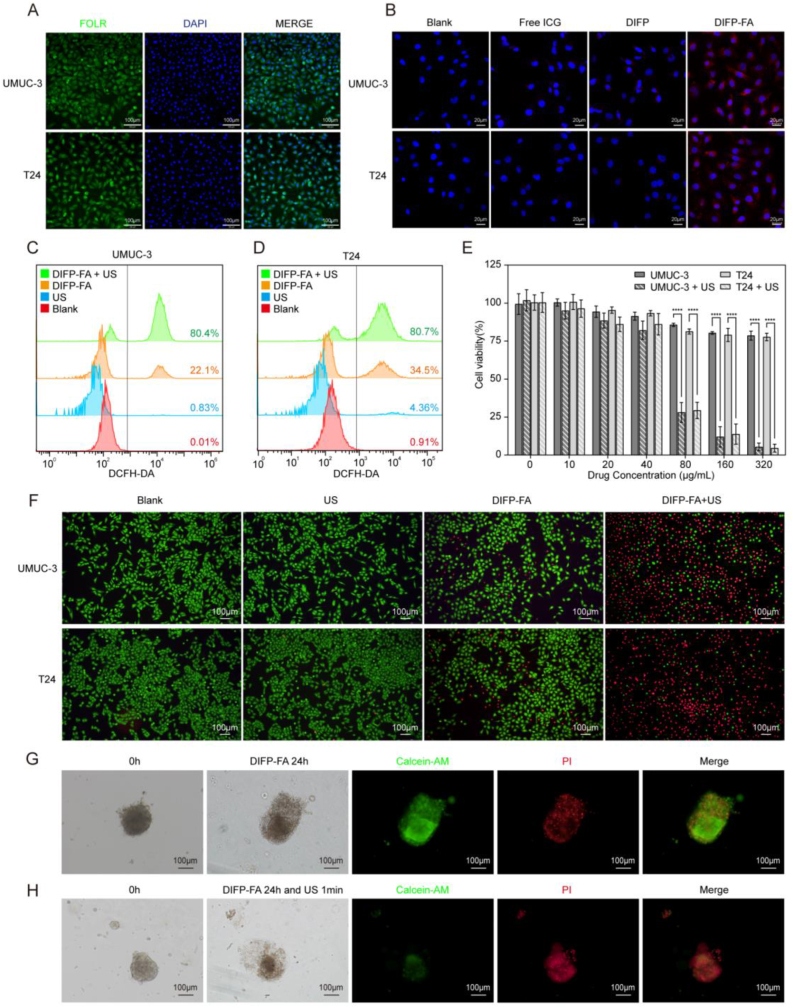
Cellular uptake of DIFP-FA and synergistic therapy toxicity in BC cells and PDOs by DIFP-FA. (A) CLSM images of FOLR expression in UMUC-3 and T24 cell lines; green: FOLR, blue: nucleus, scale bar = 100 μm. (B) CLSM images of free ICG, DIFP, and DIFP-FA after 4 h of co-incubation with UMUC-3 and T24 cells; red: ICG, blue: nucleus, scale bar = 20 μm. Flow cytometric assessment of ROS production in UMUC-3 (C) and T24 (D) cells following different treatments using DCFH-DA. (E) Cell viability of UMUC-3 and T24 cells co-incubated with various concentrations of DIFP-FA for 4 h with or without US stimulation. (F) Fluorescence microscopy images of UMUC-3 and T24 cells stained with Calcein-AM/PI after different treatments; green: live cells, red: dead cells, scale bar = 100 μm. (G) Initial bright-field image of BC-PDOs, and bright-field and AM/PI staining images after 24 h of DIFP-FA incubation; green: live cells, red: dead cells, scale bar = 100 μm. (H) Initial bright-field images of BC-PDOs, and bright-field and AM/PI images after 24 h of DIFP-FA incubation followed by 1 min of US irradiation. (For interpretation of the references to color in this figure legend, the reader is referred to the Web version of this article.)

Intracellular ROS levels were measured using 2′,7′-dichlorofluorescin diacetate (DCFH-DA). Flow cytometry results illustrated that the proportion of high-fluorescence cells increased from 22.1 % of UMUC-3 cells and 34.5 % of T24 cells in the DIFP-FA group to 80.4 % and 80.7 % in the DIFP-FA + US group, respectively (Fig. 2C–D). Immunofluorescence further demonstrated weaker green fluorescence in the cytoplasm of the DIFP-FA group, while the DIFP-FA + US group exhibited the strongest green fluorescence compared with the blank and US groups in UMUC-3 and T24 cells (Fig. S9), illustrating the highest generation of cytotoxic ROS.

### 3.3 Cellular and PDOs toxicity by US-excited DIFP-FA

After 4 h of incubation with UMUC-3 and T24 cells at varying concentrations of DIFP-FA, as illustrated in Figs. 2E and 80 μg/mL of DIFP-FA reduced cell viability to approximately 80 % (UMUC-3: 85.7 %, T24: 81.3 %). However, when combined with US irradiation, 80 μg/mL of DIFP-FA significantly decreased cell viability to about 29 % (UMUC-3: 28.1 %, T24: 29.4 %). Considering the significant difference in cellular survival between the US-irradiated and non-US-irradiated groups (*P* < 0.0001), 80 μg/mL was determined to be the minimum effective DIFP-FA concentration for further cellular investigations. At 320 μg/mL, DIFP-FA reduced cell viability to approximately 78 % (UMUC-3: 78.6 %, T24: 77.7 %), showing that DIFP-FA alone is less cytotoxic. Ultrasound intensity at 1000 mW/cm^2^ has been shown to exert cytotoxic effects on tumor cells (Fig. S10). Therefore, in this study, ultrasound with a frequency of 1.0 MHz, a duty cycle of 20 %, and a power intensity of 500 mW/cm^2^ was selected for subsequent experiments. AM/PI staining results illustrated that, compared with the blank and US groups, the DIFP-FA group had fewer dead cells and a higher proportion of viable cells in UMUC-3 and T24 cells. The DIFP-FA + US group exhibited the most significant cell-killing effect (Fig. 2F).

In BC-PDOs, US irradiation alone resulted in no significant changes, with the PDOs maintaining intact morphology, clear boundaries, tightly packed cells, and a compact overall structure. AM/PI staining revealed minimal red fluorescence in BC-PDOs (Fig. S11). After 24 h of DIFP-FA treatment, the PDOs' membranes ruptured, previously clear structural boundaries became blurred, and cells detached from the PDOs. A clear contrast was observed between viable and dead cell regions, with the viable cell region maintaining normal structure and morphology, while the dead cell region exhibited irregular and loose cell arrangement. AM/PI staining showed a mix of red and green fluorescence within the PDOs, with a greater prevalence of red fluorescence in the detached cells (Fig. 2G). Furthermore, when PDOs were initially incubated with DIFP-FA for 24 h and then irradiated with US, the PDOs' overall structure became loose, tissue integrity was lost, and the PDOs underwent complete disintegration. Due to cell death and content leakage, the PDOs displayed uneven coloration, with dark patches and discolored areas, surrounded by significant cellular debris and remains. AM/PI staining revealed that the organoid region exhibited the highest proportion of red fluorescence, with very few green fluorescent cells (Fig. 2H).

### 3.4 US-enhanced nanocatalytic ferroptosis enabled by DIFP-FA

To elucidate the potential mechanism underlying the antitumor effect of DIFP-FA + US, transcriptome analysis of UMUC-3 cells treated with PBS or DIFP-FA + US was conducted. A total of 19,039 genes were analyzed, revealing significant differences in gene expression between the DIFP-FA + US treated group and the control group, as illustrated by the clustered heat map (Fig. 3A). The volcano plot depicted the distribution of differentially expressed genes (log FC (fold change) > 1 or < −1, *P* < 0.05) between the two groups (Fig. S12), identifying 618 differentially expressed genes, with 211 upregulated and 407 downregulated in the DIFP-FA + US group compared with the control group (Table S2). KEGG enrichment analysis (Fig. 3B) illustrated that the differentially expressed genes were enriched in the "Ferroptosis" pathway. Pathways such as "Alanine, aspartate, and glutamate metabolism" (glutamate being a precursor for GSH synthesis), "Glycine, serine and threonine metabolism" (glycine being a component of GSH), and "Cysteine and methionine metabolism" (cysteine being a direct precursor for GSH) exhibited lower Q-values, illustrating significant enrichment. This shows that DIFP-FA + US effectively influences the redox balance, including intracellular GSH levels. Furthermore, higher enrichment factors for pathways such as "Protein processing in the endoplasmic reticulum" and "Antigen processing and presentation" imply that DIFP-FA + US treatment may impact cellular immunity.

**Fig. 3:**
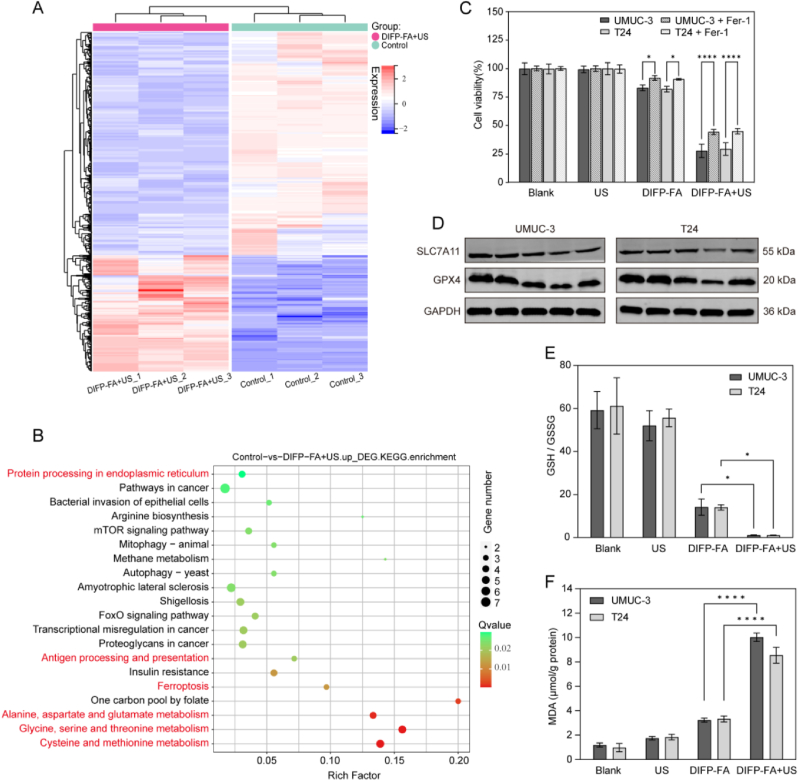
Transcriptome analysis and DIFP-FA synergistic therapy induced ferroptosis. (A) Heatmap of differentially expressed genes in UMUC-3 cells between DIFP-FA + US and PBS groups (log FC > 1 or log FC < −1, *P* < 0.05). (B) KEGG enrichment analysis highlighting the top 20 pathways enriched for differentially expressed genes between DIFP-FA + US and PBS groups. (C) Cell survival rates of UMUC-3 and T24 cells after various treatments, with or without Fer-1 (0.5 μM) (n = 3). (D) Expression levels of SLC7A11 and GPX4 in UMUC-3 and T24 cells detected by WB after different treatments, with or without Fer-1 (0.5 μM). Bands from left to right: Blank, US, DIFP-FA, DIFP-FA + US, and DIFP-FA + US + Fer-1. (E) Ratio of GSH to GSSG in UMUC-3 and T24 cells after different treatments (n = 3). (F) Intracellular MDA levels in UMUC-3 and T24 cells indicating LPO after various treatments (n = 3).

Fer-1 (a ferroptosis inhibitor) was added to the culture medium of UMUC-3 and T24 cells to verify whether DIFP-FA combined with US irradiation induces ferroptosis in tumor cells. Fig. 3C shows that in the DIFP-FA + US group, the cell survival rates of UMUC-3 and T24 after the addition of Fer-1 (UMUC-3: 44.25 %, T24: 44.85 %) were higher than those of the corresponding group without Fer-1 (UMUC-3: 27.71 %, T24: 29.35 %) (*P* < 0.0001). WB analysis revealed that the expression levels of SCL7A11 (a protein inhibiting ROS-induced ferroptosis) and GPX4 (an enzyme preventing ferroptosis) decreased following DIFP-FA treatment and were further suppressed by DIFP-FA + US treatment. However, the expression of SCL7A11 and GPX4 rebounded after the addition of Fer-1 relative to the DIFP-FA + US group (*P* < 0.01) (Fig. 3D and Fig. S13). Additionally, ROS generated by DIFP-FA + US can deplete GSH in tumor cells, converting GSH to GSSG. As shown in Fig. 3E, the GSH/GSSG ratio decreased after co-incubation of UMUC-3 and T24 cells with DIFP-FA and further declined in the DIFP-FA + US group (*P* < 0.01). The reduced levels of GSH and GPX4 can lead to cellular LPO, a typical biomarker of ferroptosis. The MDA assay demonstrated that UMUC-3 and T24 cells in the DIFP-FA + US group produced a significant amount of LPO (Fig. 3F).

### 3.5 DIFP-FA with US promotes the translocation and release of DAMPs

CRT immunofluorescence results demonstrated that cells in the DIFP-FA group exhibited slight green fluorescence. However, in the DIFP-FA + US group, the plasma membrane regions of UMUC-3 and T24 cells showed intense green fluorescence, illustrating significant CRT aggregation (Fig. 4A). As illustrated in Fig. 4B, red fluorescence is predominantly localized within the nuclear compartment. In the DIFP-FA group, nuclear fluorescence decreased, while cytoplasmic fluorescence increased. In the DIFP-FA + US group, nuclear fluorescence was almost entirely absent, and cytoplasmic fluorescence also diminished. WB analysis revealed that following DIFP-FA + US treatment, CRT expression levels increased, whereas HMGB1 levels decreased in UMUC-3 and T24 cells (Fig. 4C and Fig. S14). Furthermore, HMGB1 concentration in the cell culture medium of the DIFP-FA + US group significantly increased (Fig. 4D), illustrating that HMGB1 progressively translocated from the nucleus to the cytoplasm and was subsequently released from the cell post-DIFP-FA + US treatment. Additionally, intracellular ATP content in UMUC-3 and T24 cells of the DIFP-FA + US group significantly decreased, while extracellular ATP content markedly increased, illustrating substantial ATP release induced by DIFP-FA + US (Fig. 4E–F).

**Fig. 4:**
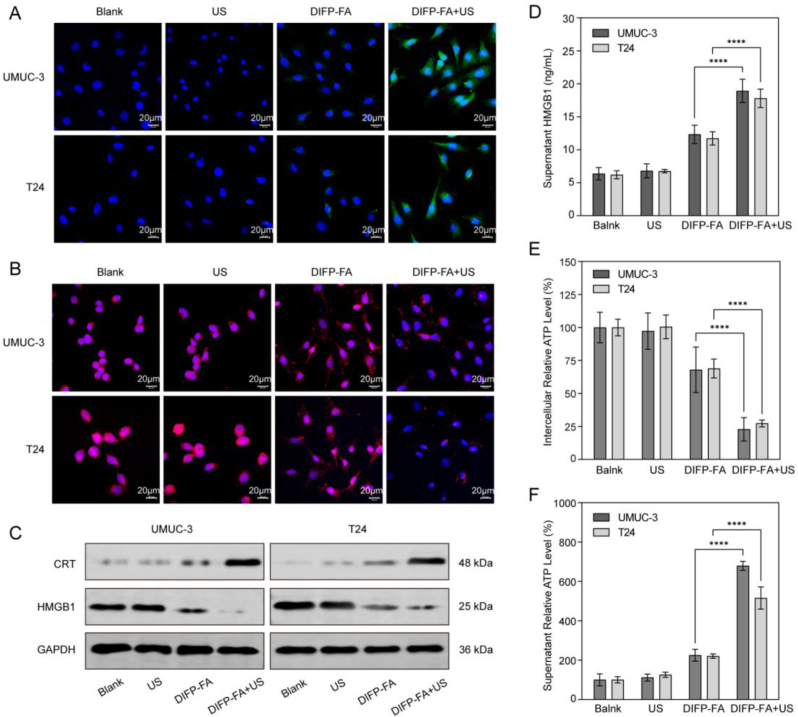
DIFP-FA synergistic therapy promotes the release of cellular DAMPs. (A) CLSM images of CRT exposure on the surface of UMUC-3 and T24 cells after different treatments; green: CRT, blue: nucleus. (B) CLSM images of HMGB1 secretion in UMUC-3 and T24 cells after various treatments; red: HMGB1, blue: nucleus. (C) Expression levels of CRT and HMGB1 in UMUC-3 and T24 cells detected by WB after different treatments. (D) Quantification of supernatant HMGB1 in UMUC-3 and T24 cells following various treatments, conducted through ELISA. Relative expression of intracellular ATP (E) and supernatant ATP (F) in UMUC-3 and T24 cells after different treatments. (For interpretation of the references to color in this figure legend, the reader is referred to the Web version of this article.)

### 3.6 Antitumor effects of DIFP-FA with US *via* ferroptosis and ICD *in vivo*

H&E and IHC staining of PDX tumors at two passages revealed similar histological features, Uroplakin II (UPK II) staining patterns, and high FOLR expression as observed in the primary tumors (Fig. S15). The *in vivo* antitumor experiment procedure is depicted in Fig. 5A. At the end of the experimental period, a significant reduction in tumor volume and weight was observed in the DIFP-FA + US group compared with the saline, US, and DIFP-FA groups (Fig. 5B–C). Fig. 5D illustrated rapid tumor volume growth in the saline and US groups during the experimental period, while tumor volume growth was inhibited in the DIFP-FA group. The tumor growth inhibition index (TGI) of the DIFP-FA + US group was significantly higher than that of the DIFP-FA group (86.4 % vs 35.6 %). The absence of weight loss in all mouse groups during the entire experimental period illustrates that DIFP-FA combined with US does not adversely affect mouse growth (Fig. 5E). IHC staining revealed that tumor tissues in the DIFP-FA + US group exhibited the lowest levels of GPX4, SLC7A11, and Ki-67 staining (Fig. 5F), illustrating that DIFP-FA + US induces ferroptosis and reduces tumor proliferation. In the DIFP-FA + US group, tumor tissues exhibited significantly enhanced CRT fluorescence, while nuclear HMGB1 staining intensity decreased (Fig. 5F). This shows that damaged tumor cells effectively secrete DAMPs.

**Fig. 5:**
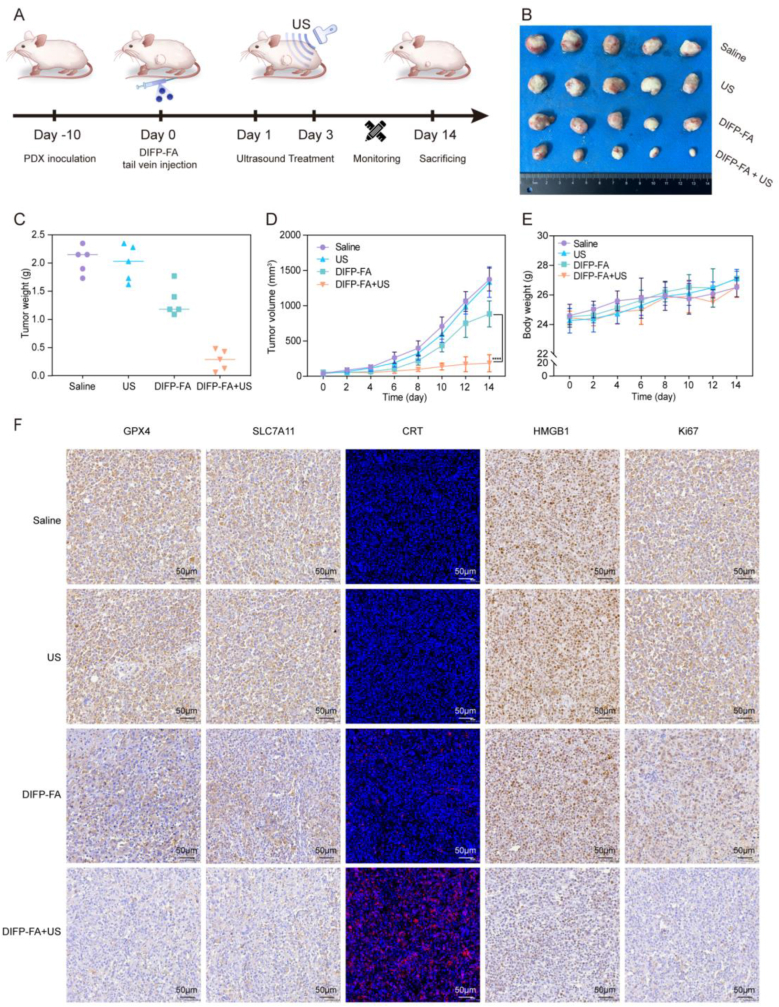
Antitumor effects of DIFP-FA synergistic therapy *via* ferroptosis and ICD *in vivo*. (A) Flowchart of US-stimulated DIFP-FA treatment for BC-PDX tumors. Digital images (B) and weights (C) of BC-PDX tumor tissue harvested from each group at the end of the experiment (n = 5). Line graph depicting BC-PDX tumor sizes (D) and NCG mice body weights (E) throughout the experimental period (n = 5). (F) Immunohistochemistry or immunofluorescence images of GPX4, SLC7A11, CRT, HMGB1, and Ki67 in BC-PDX tumor sections at the experiment's conclusion, scale bar = 50 μm.

### 3.7 DIFP-FA with US promotes immune cell infiltration and enhances ICB *in vivo*

Approximately one week post-hPBMC tail vein injection into NCG mice, white blood cell levels, lymphocyte counts, and lymphocyte percentages in the blood significantly increased to normal ranges (Fig. S16), illustrating successful immune reconstitution. The experimental procedure is illustrated in Fig. 6A. After 7 days of treatment and 14 days of monitoring, the DIFP-FA + US + PD-1 group exhibited the smallest tumor volume and weight compared with the saline, PD-1, and DIFP-FA + US groups (Fig. 6B–C). Tumor volume was significantly reduced during the experimental period in the DIFP-FA + US + PD-1 group (Fig. 6D). Throughout the experimental period, no significant changes in mouse body weight were observed for any treatments, illustrating negligible adverse effects on growth (Fig. 6E). ELISA revealed elevated TNF-α and INF-γ levels in tumor tissues within the DIFP-FA + US + PD-1 group (Fig. 6F–G). Histological examination of mouse tumor tissues showed structurally dense tumor tissues in the DIFP-FA + US group, with necrotic areas still observable (Fig. S17A). In contrast, the central structure of the tumor tissue in the DIFP-FA + US + PD-1 group exhibited a loosening of its composition, reduced staining intensity, and decreased cell density (Fig. S17B). IHC staining for CD4 and CD8 in tumor tissues revealed almost no positive staining in the saline and PD-1 groups. Conversely, the DIFP-FA + US group showed stronger staining with CD4 and CD8-positive T-lymphocyte aggregates in necrotic areas. The DIFP-FA + US + PD-1 group exhibited the strongest CD4 and CD8 staining in necrotic areas and margins, with the greatest infiltration of CD4-positive T lymphocytes upon local magnification (Fig. 6H–I).

**Fig. 6:**
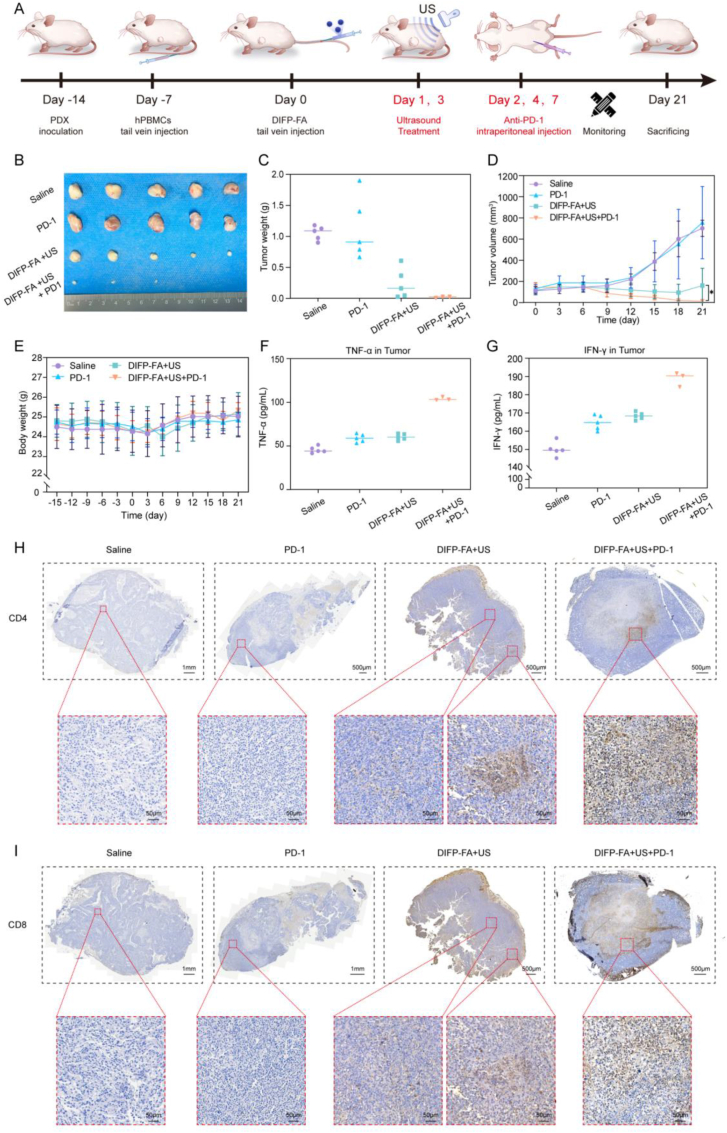
DIFP-FA synergistic therapy promotes immune cell infiltration and enhances ICB *in vivo*. (A) Flowchart of DIFP-FA + US combination anti-PD-1 tumor therapy. Digital images (B) and weights (C) of BC-PDX tumors harvested from each group at the experiment's conclusion (n = 5). (D) Line graph depicting BC-PDX tumor sizes within 14 days of treatment initiation (n = 5). (E) Line graph showing body weights of NCG mice from the time of hPBMC tail vein injection to the experiment's end (n = 5). Quantification of TNF-α (F) and IFN-γ (G) in PDX tumor tissue at the end of the experiment. IHC images of CD4 (H) and CD8 (I) in tumor tissues at the experiment's conclusion. Top row: general view of whole tumor tissue (scale = 1 mm or 500 μm), Bottom row: local magnification of corresponding tumor tissue (scale = 50 μm).

### 3.8 Biodistribution and biosafety of DIFP-FA

Small animal fluorescence imaging post-DIFP-FA intravenous injection showed the fluorescent signal initially appearing in the liver, significantly increasing and spreading to the gastrointestinal tract region after 12 h. The signal was strongest at 24 h and declined overall at 48 h, with a significant decrease in the liver region. Tumor tissues showed significant fluorescent signals after 12 h, peaking at 24 h, and decreasing at 48 h (Fig. 7A–B). These results illustrate selective accumulation of DIFP-FA within BC-PDX tumors, reaching maximum levels 24 h post-injection. Fluorescence images of major organs and tumor tissues at 24 and 48 h post-injection demonstrated strong signals in tumor tissue and liver, with no significant fluorescence in the spleen, lungs, heart, and kidneys (Fig. 7C). The liver's fluorescence signal at 48 h was weaker than at 24 h, while the tumor's fluorescence intensity remained high (Fig. 7B–C). These findings show DIFP-FA retention in tumors for an extended period.

**Fig. 7:**
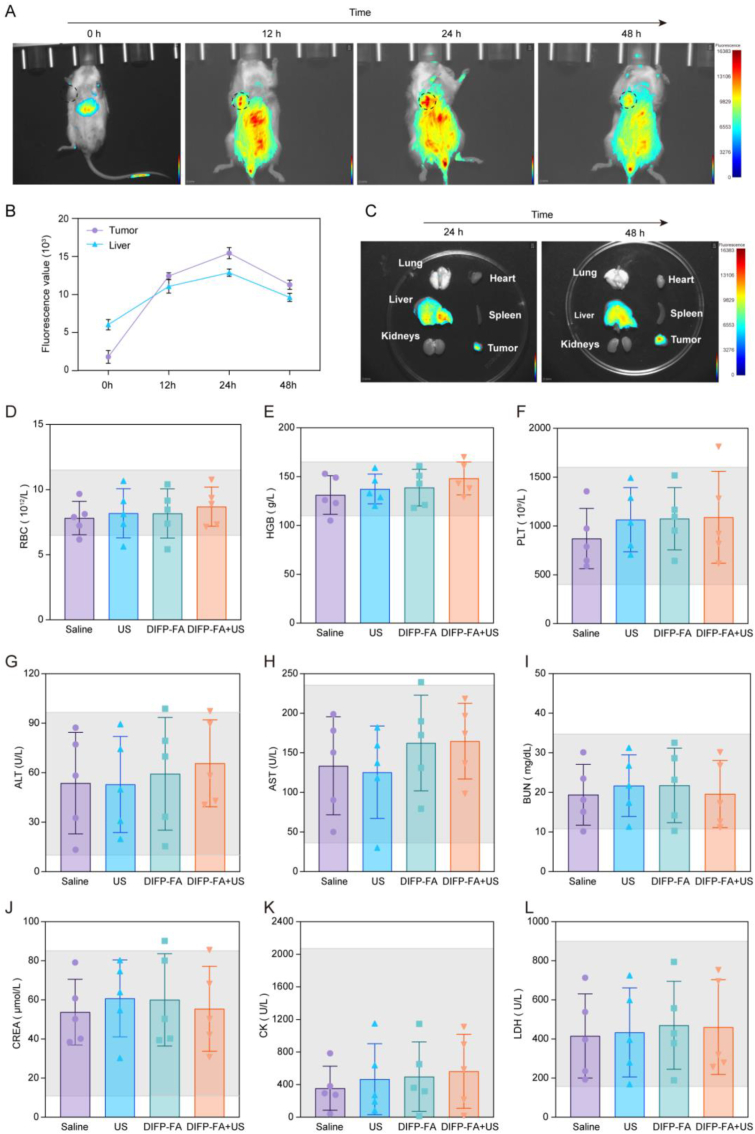
Biodistribution and biosafety of DIFP-FA. (A) Real-time fluorescence images of BC-PDX-loaded mice at 0, 12, 24, and 48 h after tail vein injection of DIFP-FA. Tumor sites are circled with black dashed lines. (B) Quantitative analysis of mean fluorescence intensity in the liver and tumors of BC-PDX-loaded mice at different time points. (C) Ex vivo fluorescence images of the lungs, heart, liver, spleen, kidneys, and tumor 24 and 48 h after tail vein injection of DIFP-FA in BC-PDX-loaded mice. (D–L) Blood biochemical tests in BC-PDX-loaded mice after 14 days of various treatments (n = 5), including RBC (D), HGB (E), PLT (F), ALT (G), AST (H), BUN (I), CREA (J), CK (K), and LDH (L)). The grey area is the normal range of the indicator.

Blood tests of the DIFP-FA + US group at the end of the experiment showed that hematological parameters (RBC, HGB, PLT), liver function markers (AST, ALT), renal function markers (BUN, CREA), and myocardial function markers (CK, LDH) were within physiological ranges (Fig. 7D–L). Despite using severely immunodeficient mice with low leukocyte and lymphocyte counts (Fig. S18), no nonexperimental deaths occurred throughout the experimental cycle. H&E staining of major organs (heart, kidney, liver, lung, intestine, spleen, and skin) revealed no tissue necrosis, illustrating that DIFP-FA does not exhibit significant biotoxicity (Fig. S19).

## 4 Discussion

BC treatments, including chemotherapy and immunotherapy, suffer from limited efficacy and severe side effects, contributing to a suboptimal cure rate [2,3,11]. Tumor is a complex disease that involves a multitude of mechanisms and pathways. Therefore, the therapeutic response rate of a single treatment modality is limited. The combination of various nanomedicines with chemotherapy, PDT, SDT, or immunotherapy has emerged as a promising strategy to enhance antitumor efficacy and improve cancer treatment outcomes [[12], [13], [14]]. Our synthesized DIFP-FA targets BC *via* FA for precise drug delivery and can be combined with US to achieve spatiotemporally controlled drug release, thereby enhancing bioavailability. DIFP-FA integrates multiple treatment modalities such as chemotherapy and SDT/CDT for the effective treatment of BC.

The size variations of drug delivery systems within the complex human internal environment are crucial for their effective delivery to tumor sites [15]. Larger-sized drug delivery systems (100–200 nm) extend circulation time but often fail to penetrate deeply into tumor tissues, while smaller-sized systems (4–20 nm) can easily traverse tumor tissue but may exhibit inadequate drug retention [16]. This study demonstrated that the initial size of DIFP-FA was approximately 215 nm, with DOX release increasing in acidic environments. This illustrates that DIFP-FA can prolong circulation time in the bloodstream and reduce its size in response to the acidic environment of solid tumors, thereby enhancing drug retention. Our previous findings revealed that the ester and amide bonds in the PPPs constituting the DIFP-FA shell degrade at pH below 6.5, leading to size contraction and eventual hydrolysis of DIFP-FA [6]. Additionally, DIFP-FA's negative zeta potential allows it to remain stable and dispersed in various physiological solutions. Nanoparticles with negative zeta potential avoid adhesion to negatively charged proteins and prolong blood circulation time [17]. Therefore, DIFP-FA is expected to remain stable in the blood for extended periods, with contraction and degradation within the acidic TME ensuring efficient tumor penetration and controlled release of DOX and ICG.

The FA surface modification of DIFP-FA exhibits specific binding to FOLR on tumor cells, facilitating targeted delivery to cells with high FOLR expression and enhancing intracellular drug uptake [6]. This study found that BC cells universally exhibited high FOLR expression. Compared with non-targeted drugs such as DIFP and free ICG, FA modified on the surface of DIFP-FA can specifically bind to FOLR, thereby enhancing the uptake of DIFP-FA by BC cells. *In vivo* experiments confirmed that DIFP-FA specifically accumulated in BC-PDX tumor tissues, enabling targeted delivery and efficient cargo enrichment. Thus, targeting FOLR in BC may be a key strategy for achieving composite multifunctional drug-targeted therapy.

Previous research demonstrated that DIFP-FA can respond to NIR and TME, enhancing tumor accumulation and improving therapeutic efficacy [6]. However, PDT encounters several limitations in clinical tumor treatment. Firstly, the NIR light used in PDT penetrates to a limited depth (<1 cm) [7], restricting its effectiveness to skin and superficial tissues, and rendering it ineffective for deep tumors. Secondly, systemic administration of photosensitizers can lead to skin photosensitization reactions, potentially causing severe skin damage. Lastly, light absorption and scattering in tissues result in energy attenuation, limiting PDT's application for larger or deeper tumors. Conversely, US offers superior tissue penetration, effectively reaching deep-seated tumors with minimal energy attenuation and reduced impact on surrounding normal tissues, thus enhancing treatment safety. Consequently, US-activated drugs present a more suitable approach for clinical treatment. SDT, utilizing US to activate sonosensitizers, has shown considerable antitumor potential [9,18,19]. In this study, *in vitro* experiments demonstrated that ICG loaded in DIFP-FA generates ^1^O_2_ upon US stimulation, Fe/FeO NCs in the DIFP-FA shell produce ·OH *via* the Fenton reaction, and DOX encapsulated in DIFP-FA intercalates into DNA, inhibiting DNA synthesis, leading to direct tumor cell death. In the present study, DOX, delivered *via* DIFP-FA, plays a significant role in the inhibition of tumor progression. The use of DIFP-FA enhances the targeted delivery of DOX to the tumor site, thereby increasing its effective concentration and cytotoxicity. PDOs and cisplatin-gemcitabine as well as tislelizumab-resistant PDX experiments confirmed that DIFP-FA achieves significant antitumor activity by combining SDT/CDT and chemotherapy.

Transcriptome analysis was conducted to explore the potential antitumor mechanism of DIFP-FA + US. KEGG enrichment analysis identified pathways related to ferroptosis and GSH metabolism. Ferroptosis, a form of programmed cell death dependent on iron, is characterized by LPO accumulation [20]. Upon uptake by tumor cells, the iron in DIFP-FA induces intracellular iron overload, catalyzing the generation of highly reactive ·OH from hydrogen peroxide. Additionally, ICG produces ^1^O_2_ in response to US stimulation, further increasing intracellular oxidative load. This study demonstrated that ROS generated by DIFP-FA + US deplete GSH, converting it to GSSG in tumor cells, ultimately leading to LPO. Moreover, SLC7A11 and GPX4 are key targets for ferroptosis. SLC7A11, a component of System Xc^−^, mediates extracellular cysteine uptake and intracellular glutamate export, and its inhibition reduces GSH synthesis [21]. GPX4, a GSH-dependent enzyme, reduces lipid peroxides, protecting cell membranes from oxidative damage [22]. This study showed that DIFP-FA + US impaired SLC7A11 and GPX4 function in BC cells and BC-PDX tumors. Conversely, adding the ferroptosis inhibitor Fer-1 restored SLC7A11 and GPX4 expression in BC cells, thereby restoring cell viability. DIFP-FA + US induces ferroptosis by generating ROS, which leads to lipid peroxidation and cell death. Ferroptosis has emerged as an effective mechanism in cancer therapy, especially when combined with traditional chemotherapy agents like DOX. The cooperative effects of DOX and ferroptosis lead to a comprehensive attack on tumor cells, making DIFP-FA + US a powerful combination therapy. This synergistic approach not only enhances cytotoxicity but also circumvents resistance mechanisms, providing a promising strategy for drug-resistant tumors.

KEGG enrichment analysis underscored the importance of the "Protein processing in endoplasmic reticulum" and "Antigen processing and presentation" pathways, which are intricately linked to endoplasmic reticulum stress and ICD respectively. In this study, DIFP-FA + US induced ICD by promoting CRT exposure and releasing HMGB1 and ATP in BC cells and BC-PDX tumors. CRT exposure on the cell surface functions as an "eat me" signal, initiating the immune response by binding to cells expressing CD91, thereby recruiting antigen-presenting cells (APCs), facilitating antigen presentation, and triggering the release of pro-inflammatory cytokines [23]. Additionally, HMGB1 released by cells promotes the activation and migration of DCs and stimulates the release of pro-inflammatory factors by engaging pattern recognition receptors such as Toll-like receptor 4 [24]. Extracellular ATP released by dying tumor cells acts as a "find me" signal, mediating DCs recruitment, activation, and inflammatory responses through binding to P2RX7 purinergic receptors on DCs [25]. DAMPs such as CRT, HMGB1, and ATP, released or exposed during ICD, enhance APCs function, thereby amplifying anti-tumor immune responses [26].

DIFP-FA + US induced ferroptosis and ICD in BC-PDX tumor tissues, actively releasing and exposing various DAMPs. This experiment further constructed a humanized BC-PDX model with hPBMCs to evaluate the immune activation effect of DIFP-FA under US action. Results demonstrated that DIFP-FA + US not only effectively eradicated PDX tumor tissue but also stimulated and enhanced APCs function by releasing DAMPs, leading to the recruitment and infiltration of CD4^+^ T helper cells and CD8^+^ cytotoxic T cells into the tumor tissue. Furthermore, TNF-α and IFN-γ levels significantly increased locally in tumors treated with DIFP-FA + US + PD-1. TNF-α and IFN-γ are pivotal immune cytokines that directly inhibit tumor cell growth, enhance immune cell cytotoxic function, and modify the TME to facilitate immune cell infiltration and attack [27,28]. DIFP-FA + US triggers immune activation, increasing CD4^+^ and CD8^+^ cell infiltration within the tumor tissue, while the anti-PD-1 antibody alters the inhibitory TME, enabling the immune system to more effectively recognize and eliminate tumor cells, thus achieving effective clearance of cisplatin-gemcitabine and tislelizumab-resistant BC.

DIFP-FA leverages FA to achieve targeted uptake into tumor cells. Its unique properties, responsive to TME and US, ensure that it exerts potent therapeutic effects solely within tumor tissue under US irradiation, thereby minimizing damage to healthy tissue. Compared with systemic chemotherapeutic agents and the unavoidable skin phototoxicity associated with PDT, DIFP-FA targeted delivery of chemotherapeutic drugs combined with SDT/CDT exhibits superior biosafety while effectively eradicating tumor tissue. DIFP-FA integrates chemotherapy, SDT/CDT-induced ferroptosis, and ICD in BC, igniting immune responses within the TME and enhancing the efficacy of ICB therapy. For tumors resistant to chemotherapy and immunotherapy, multifunctional drugs may become an ideal option and a focus for future research. This study offers a safe and reliable therapeutic approach for the clinical treatment of BC, particularly for cases unresponsive to ICB therapy. However, the complex synthesis method and stringent conditions required for DIFP-FA hinder mass production, and the underdevelopment of SDT-related medical devices obstructs clinical translation. Furthermore, although BC cells highly express FOLR, it is also present in human alveoli and renal proximal tubules [29], potentially causing side effects when applied to humans. Future research should focus on simplifying drug synthesis methods and identifying BC receptors with higher specificity, thereby improving the efficiency and targeting of drug synthesis. Furthermore, future research will also focus on a comprehensive analysis of the effects of DIFP-FA + US therapy on gene expression within PDX tumor tissues. The objective is to delineate its impact on tumor progression, immune evasion, and drug resistance in a complex three-dimensional tumor context. These insights are anticipated to facilitate the optimization of combined therapeutic modalities and targeted treatment strategies, thereby expediting their clinical translation.

## 5 Conclusion

The composite multifunctional targeted drug DIFP-FA integrates SDT/CDT with chemotherapy for potent tumor eradication and immune activation. This synergistic approach induces ferroptosis and ICD, enhancing antitumor immunity and promoting T lymphocyte infiltration. Combined with PD-1 checkpoint blockade therapy, it disarms the immunosuppressive TME, effectively killing tumors resistant to immunotherapy. This US-enhanced nanocatalytic therapy, paired with PD-1 inhibition, offers valuable insights for developing targeted drugs against immunotolerant tumors and holds significant clinical translational potential.

## CRediT authorship contribution statement

**Yibo Shi:** Writing – review & editing, Writing – original draft, Visualization, Validation, Software, Methodology, Investigation. **Guangrui Fan:** Writing – review & editing, Data curation. **Enguang Yang:** Project administration, Methodology. **Yuanfeng Zhang:** Visualization, Validation. **Hui Ding:** Funding acquisition, Formal analysis. **Junqiang Tian:** Investigation, Funding acquisition, Formal analysis. **Liang Cheng:** Writing – review & editing, Supervision, Conceptualization. **Hanzhang Wang:** Supervision, Resources. **Tianzhi Hao:** Investigation, Data curation. **Baodui Wang:** Writing – review & editing, Supervision, Conceptualization. **Zhiping Wang:** Writing – review & editing, Supervision, Project administration, Funding acquisition, Conceptualization.

## Ethics approval and consent to participate

This study has been performed in accordance with the Declaration of Helsinki and approved by the Ethics Committee of the Second Hospital of Lanzhou University (approval numbers: D2023-059 and 2023A-043). Written informed consent was obtained from each patient. All patient data were kept anonymous. Animal experiments adhered to the National Institutes of Health guidelines for the Care and Use of Laboratory Animals and the Regulations for the Administration of Affairs Concerning Experimental Animals (2017.03.01 edition) published by the State Council of the People's Republic of China. At the experiment's conclusion, animals were euthanized by an overdose of inhalation anesthetics.

## Consent for publication

Not applicable.

## Availability of data and materials

The datasets used and/or analyzed during the current study are available from the corresponding author on reasonable request.

## Funding

The National 10.13039/501100001809Natural Science Foundation of China (Grant No.051160007).

The Major Science and Technology Project of Gansu Province (Grant No. 24ZDFA002).

## Declaration of competing interest

The authors declare that they have no known competing financial interests or personal relationships that could have appeared to influence the work reported in this paper.

## Figures and Tables

**figs1:**
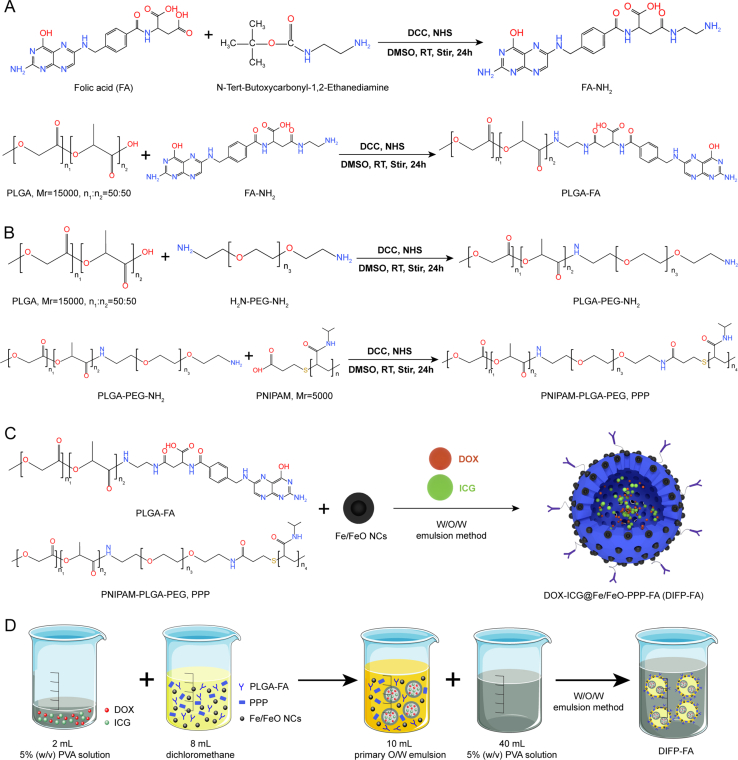
Detailed synthesis process and schematic diagram of DIFP-FA. The schematic diagram was created using Servier Medical Art (https://smart.servier.com). Servier Medical Art by Servier is licensed under a Creative Commons Attribution 4.0 Unported License (https://creativecommons.org/licenses/by/4.0/).

**figs2:**
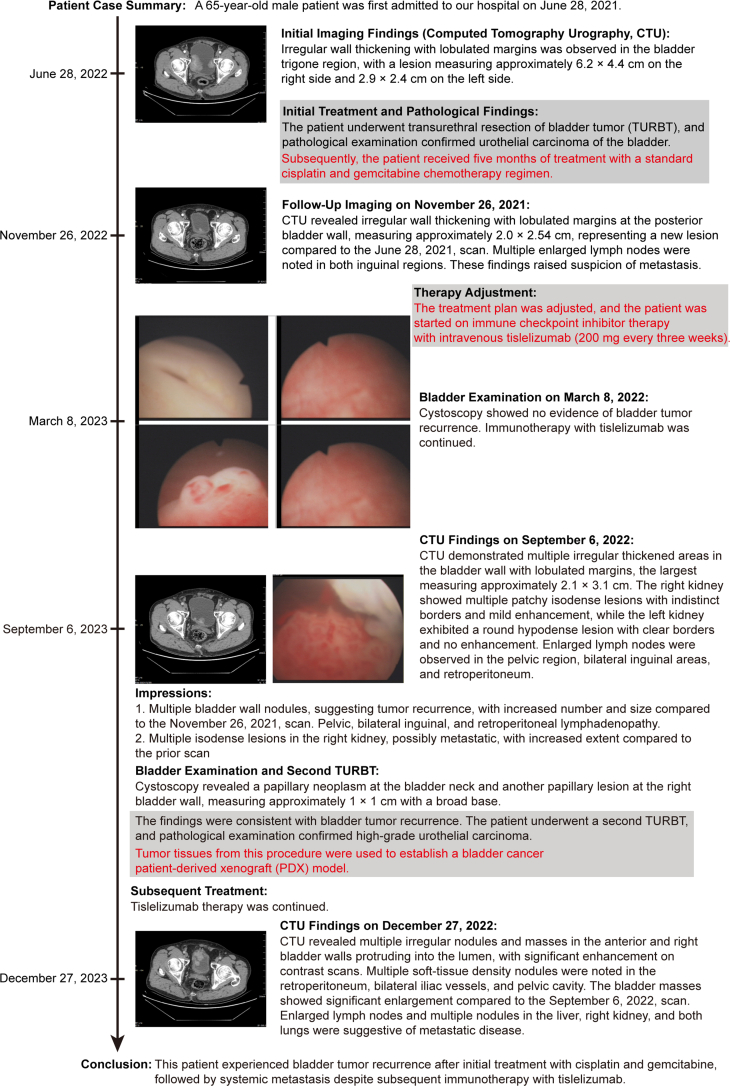
The diagnosis and treatment of a patient with BC who exhibited resistance to both cisplatin and gemcitabine, as well as recurrence following tislelizumab treatment.

**figs3:**
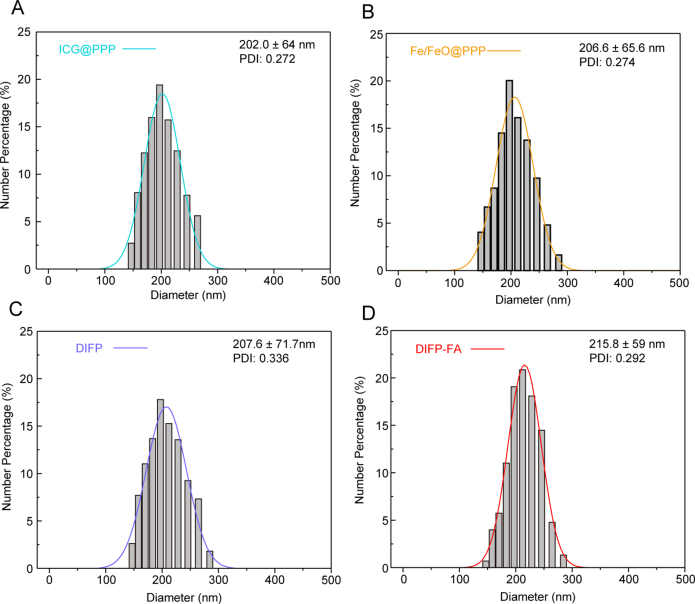
Particle size distribution of NPs at different steps in the synthesis of DIFP-FA measured by DLS. (A) ICG@PPP (202.0 ± 64 nm, PDI = 0.727), (B) Fe/FeO@PPP (206.6 ± 65.6 nm, PDI = 0.274), (C) DIFP (207.6 ± 71.7 nm, PDI = 0.336), (D) DIFP-FA (215.8 ± 59 nm, PDI = 0.292). Related to Figure 1C.

**figs4:**
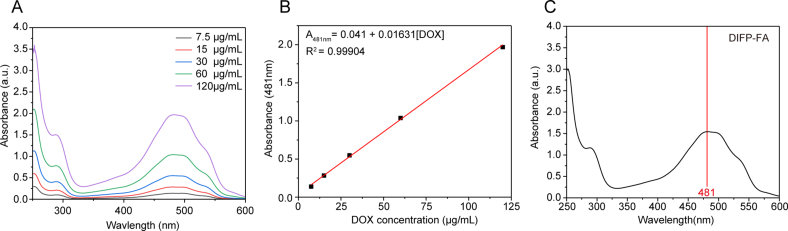
Detection of DOX encapsulation rate in DIFP-FA. (A) UV-Vis absorption spectra of different concentrations of DOX, (B) The absorbance at 481 nm was selected to plot the DOX concentration-absorbance standard working curve, (C) UV-visible spectra of DOX released from 5 mg of DIFP-FA

**figs5:**
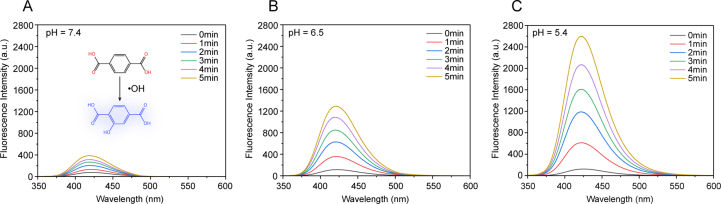
Detection of ·OH generated by DIFP-FA using TA. Fluorescence spectra of TAOH with increasing exposure time to DIFP-FA in (A) pH = 7.4, (B) pH = 6.5, and (C) pH = 5.4 solutions, respectively. Inset in (A): After the oxidation of terephthalic acid (TA) to 2-hydroxy-terephthalic acid (TAOH) by ·OH, nonfluorescent TA was converted to fluorescent TAOH. Related to Figure 1I.

**figs6:**
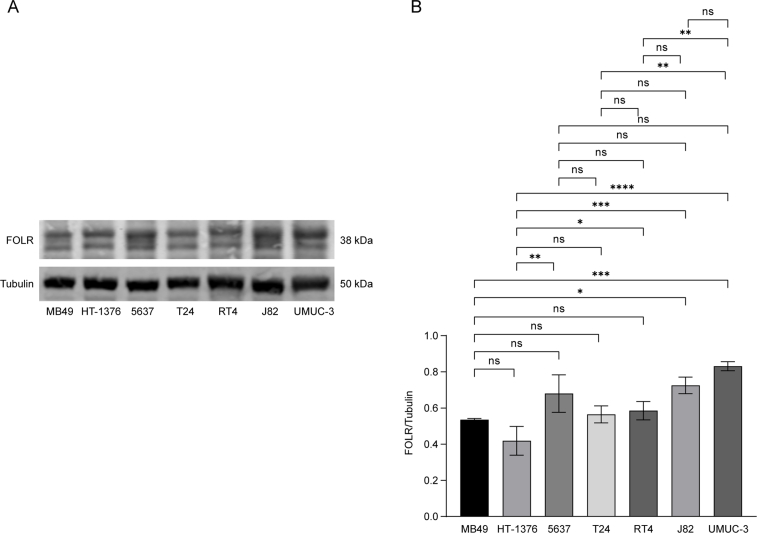
WB and quantification of FOLR in different BC cell lines. (A) Expression levels of FOLR and Tubulin in BC cell lines MB49, HT-1376, 5637, T24, RT4, J82, and UMUC-3 were detected by WB; (B) The ratio of FOLR to Tubulin was quantified using ImageJ software.

**figs7:**
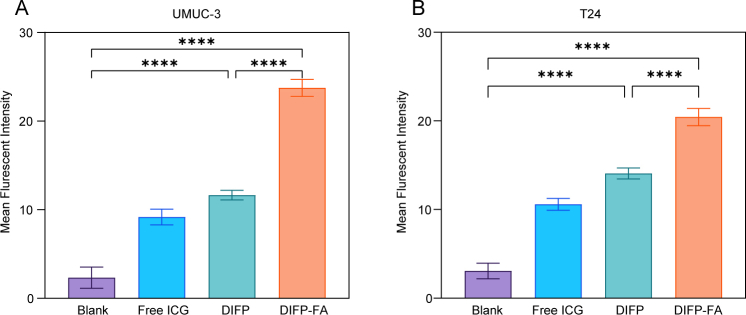
Fluorescence quantification of ICG in BC cells. Fluorescence changes of free ICG, DIFP, and DIFP-FA after 4h of co-incubation with UMUC-3 (A) and T24 (B) cells were quantified using ImageJ software. Related to Figure 2B.

**figs8:**
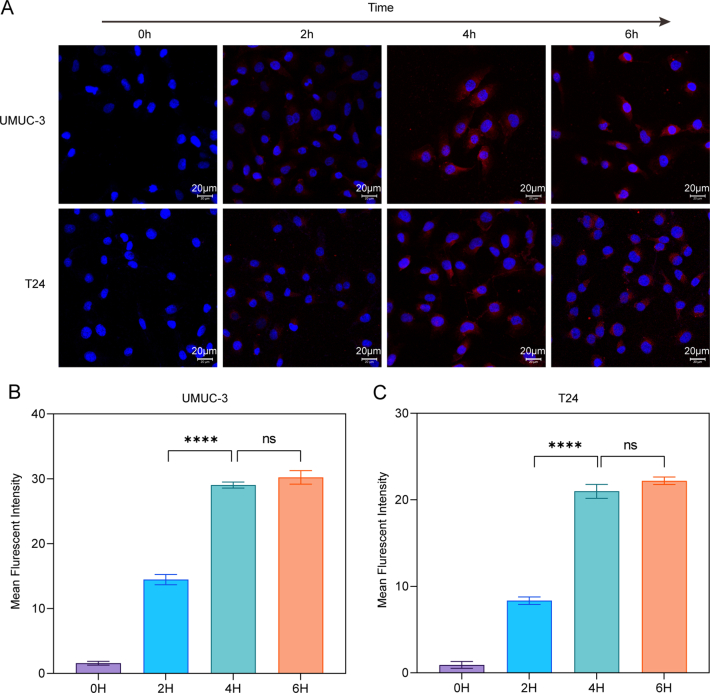
CLSM images and fluorescence quantification of BC cells with increasing DIFP-FA incubation time. (A) CLSM images of DIFP-FA after 0, 2, 4, and 6 h of co-incubation with UMUC-3 and T24 cells, scale bar = 20 μm. Fluorescence changes after incubation of DIFP-FA with UMUC-3 (B) and T24 (C) cells for 0, 2, 4, and 6 h were quantified using ImageJ software.

**figs9:**
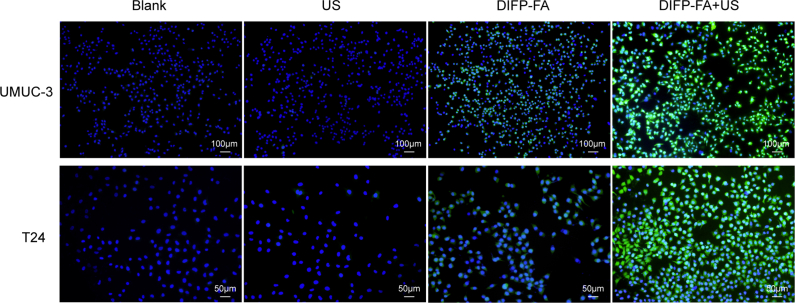
Fluorescence images of ROS production in UMUC-3 and T24 cells after different treatments detected using DCFH-DA, scale bar = 100 μm or 50 μm.

**figs10:**
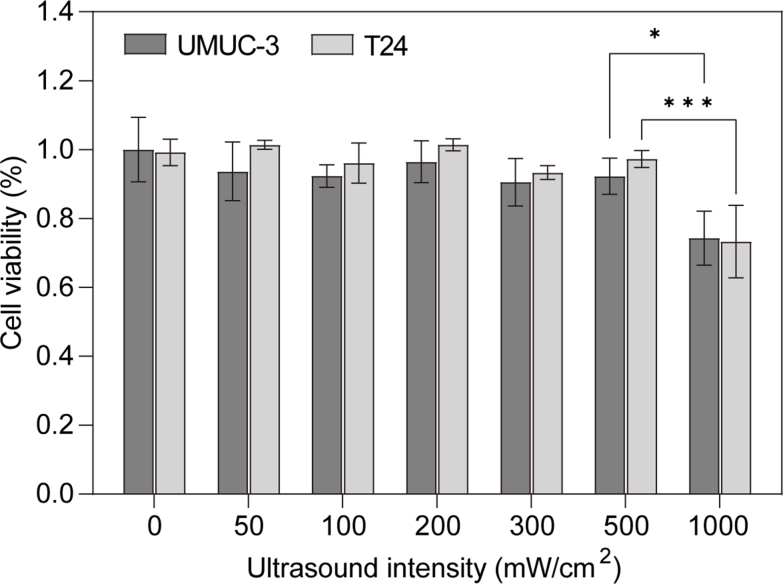
Detection of toxicity induced by US alone on BC cells. UMUC-3 and T24 cells were irradiated with US (1 MHz frequency, 20% duty cycle) at intensities of 0, 50, 100, 200, 300, 500, and 1000 mW/cm^2^ for 1 min, and cell viability was assessed by CCK8.

**figs11:**
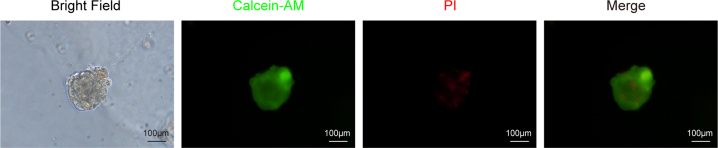
Bright field images and AM/PI staining images of BC-PDOs without any intervention, scale bar = 100 μm.

**figs12:**
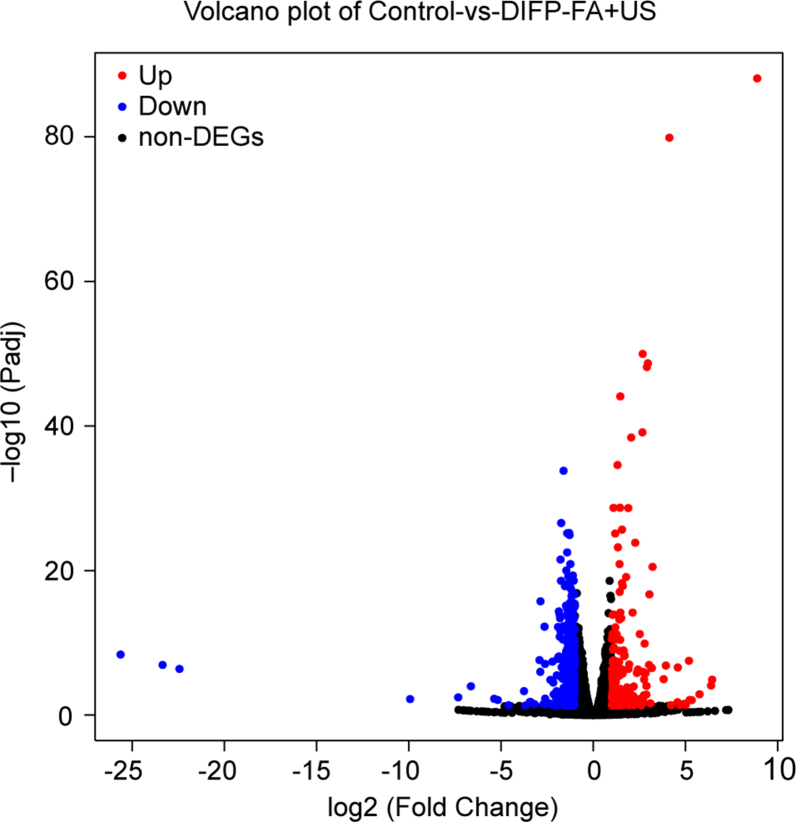
Volcano plot of differentially expressed genes up-regulated or down-regulated in the DIFP-FA + US group compared with the Control group (False Discovery Rate) < 0.05, log FC > 1, or log FC < -1).

**figs13:**
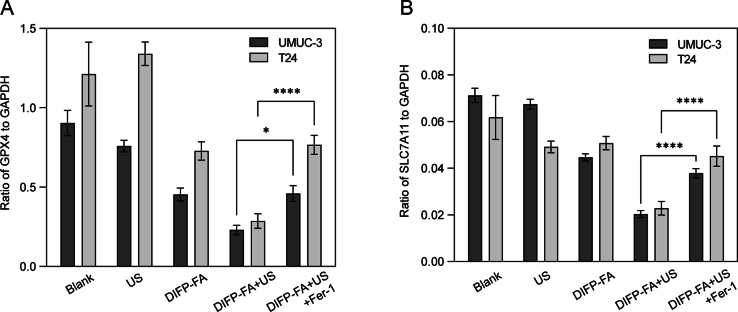
WB quantification of ferroptosis-related proteins. The ratio of GPX4 and SLC7A11 to GAPDH in UMUC-3 and T24 cells after different treatments using ImageJ software. Related to Figure 3D.

**figs14:**
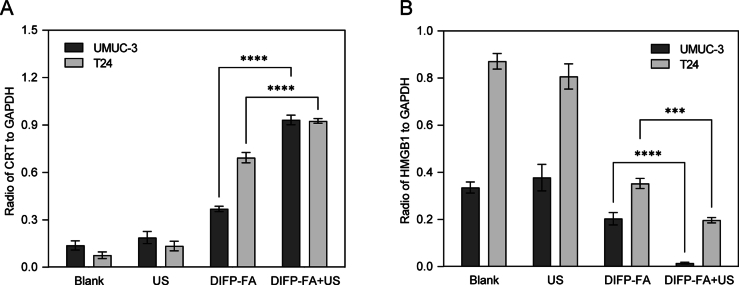
WB quantification of ICD-related proteins. The ratio of CRT and HMGB1 to GAPDH in UMUC-3 and T24 cells after different treatments using ImageJ software. Related to Figure 4C.

**figs15:**
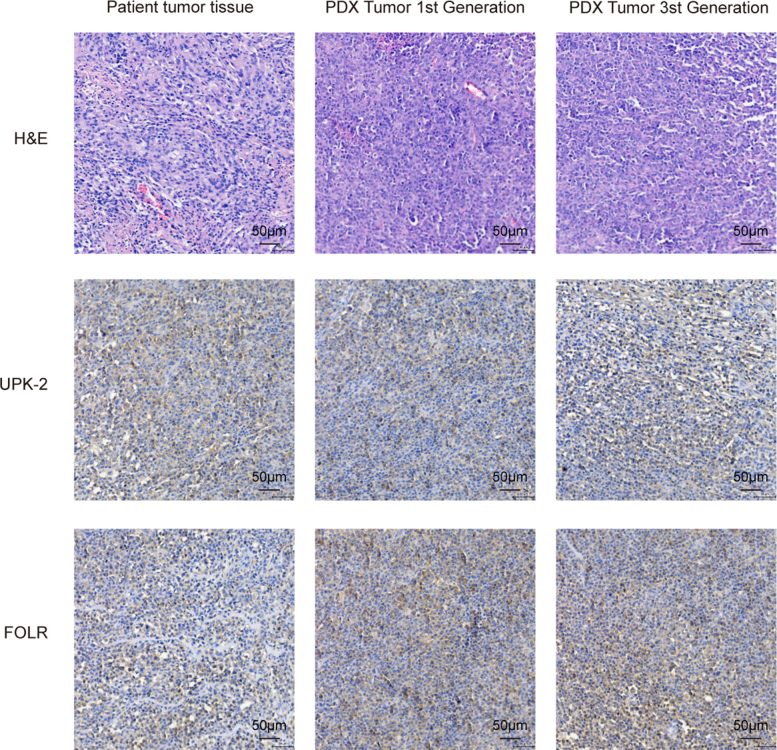
H&E staining and immunohistochemistry of the original BC tumor tissue and derived BC-PDX tumor tissue. H&E staining and immunohistochemistry images of UPK II (a key marker of uroepithelium) and FOLR in BC cancer tissues and 1st and 3rd generation BC-PDX tumor tissues prepared from them, scale bar = 50 μm.

**figs16:**
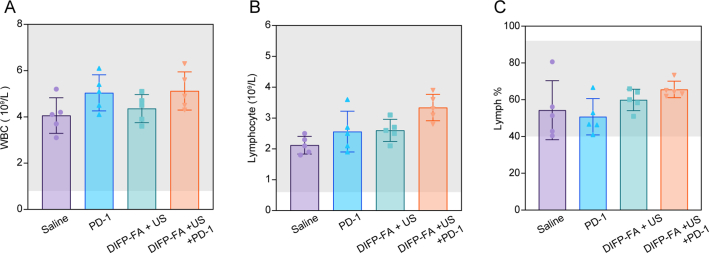
Leukocyte and lymphocyte counts in BC-PDX loaded mice 1 week after tail vein injection of hPBMCs (n = 5). The grey area is the normal range of the indicator.

**figs17:**
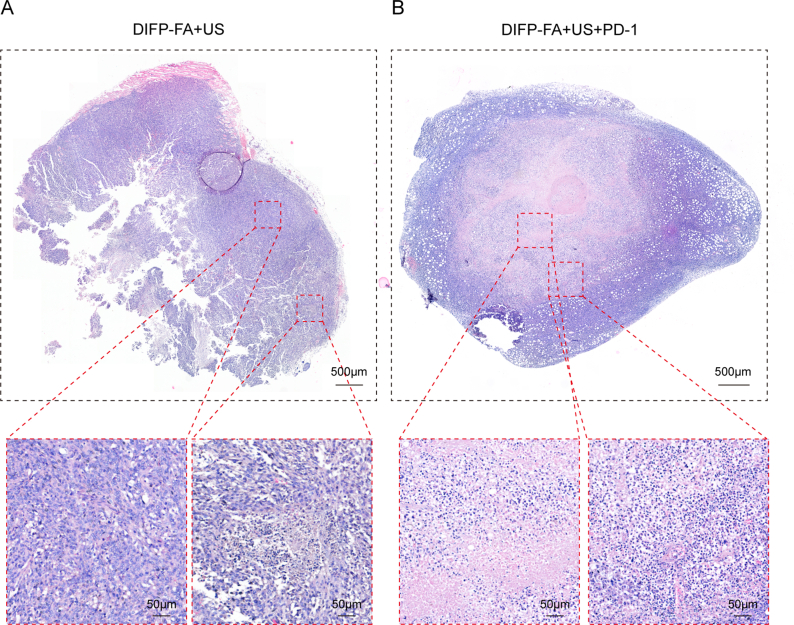
H&E staining of BC-PDX tissues treated with or without PD-1. H&E images of PDX tumor tissues in the DIFP-FA + US group and the DIFP-FA + US + PD1 group at the end of the experiment. Top row: general view of the whole tumor tissue (scale = 500 μm), Bottom row: local magnification of the corresponding tumor tissue (scale = 50 μm).

**figs18:**
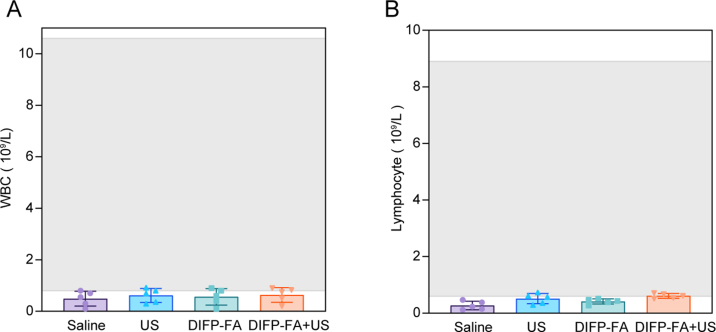
Blood WBC and lymphocyte counts in BC-PDX loaded mice from different treatment groups at the end of the 14-day experiment (n = 5). The grey area is the normal range of the indicator.

**figs19:**
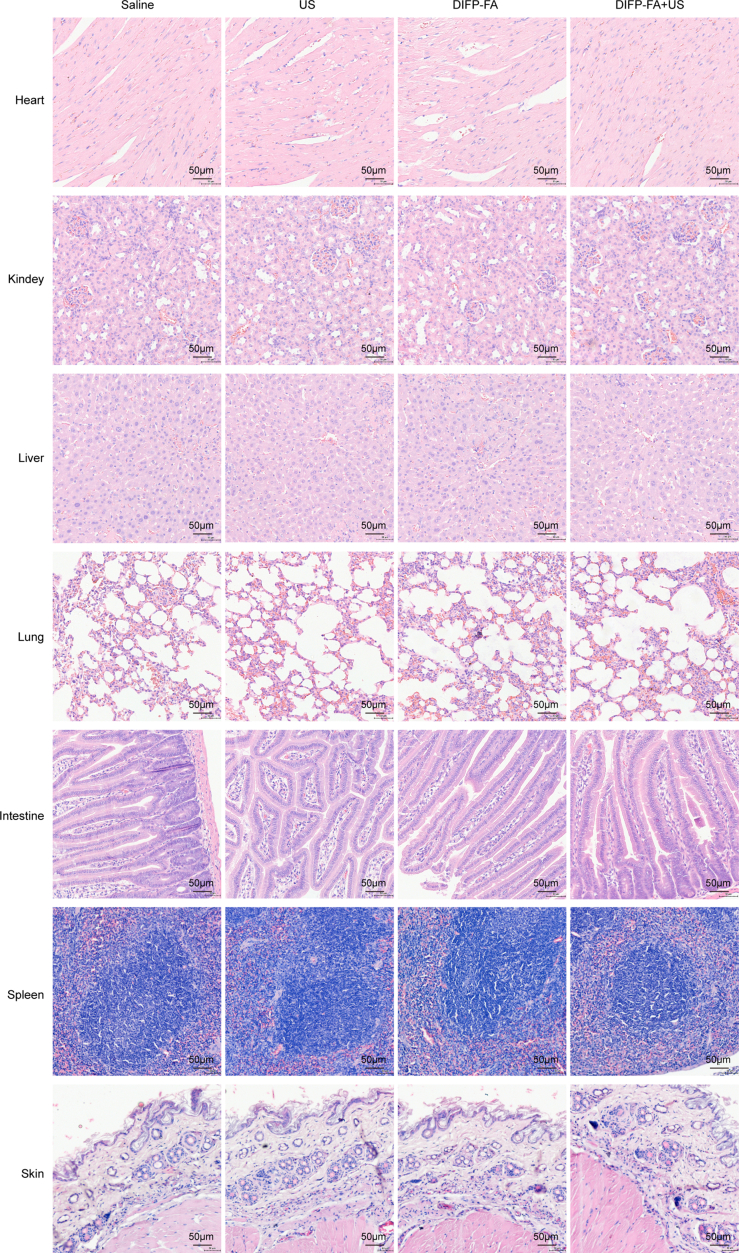
H&E stained images of major organs (heart, kidney, liver, lung, intestine, spleen, skin) of BC-PDX loaded mice from different treatment groups at the end of the experiment, scale bar = 50 μm.

## Data Availability

Data will be made available on request.

## Abbreviations

BC: bladder cancer
TURBT: transurethral resection of the bladder tumor
ICB: immune checkpoint blockade
TME: tumor microenvironment
DOX: doxorubicin
ICG: indocyanine green
NCs: nanocrystals
FA: folic acid
PPP: PNIPAM-PEG-PLGA
NIR: near-infrared
PDT: photodynamic therapy
US: ultrasound
SDT: sonodynamic therapy
PDOs: patient-derived organoids
PDX: patient-derived tumor xenograft
^1^O_2_: singlet oxygen
OH: hydroxyl radicals
CDT: chemodynamic therapy
ICD: immunogenic cell death
DAMPs: damage-associated molecular patterns
DCs: dendritic cells
SEM: scanning electron microscopy
DLS: dynamic light scattering
PBS: phosphate buffered saline
FBS: fetal bovine serum
ROS: reactive oxygen species
MB: methylene blue
DPBF: 1,3-diphenylisobenzofuran
TA: terephthalic acid
TAOH: hydroxyterephthalic acid
FOLR: folate receptor
WB: western blotting
LSCM: laser scanning confocal microscope
AM/PI: Calcein-AM and propidium iodide
EGF: epidermal growth factor
Fer-1: Ferrostatin-1
GSH: glutathione
GSSG: oxidized glutathione
LPO: lipid peroxidation
MDA: malondialdehyde
HMGB1: high mobility group box 1
CRT: calreticulin
ELISA: enzyme-linked immunosorbent assay
ATP: adenosine triphosphate
H&E: hematoxylin-eosin staining
IHC: immunohistochemistry
RBC: red blood cell
HGB: hemoglobin
PLT: platelet
AST: aspartate aminotransferase
ALT: alanine aminotransferase
BUN: blood urea nitrogen
CREA: creatinine
CK: creatine kinase
LDH: lactate dehydrogenase
hPBMCs: human peripheral blood mononuclear cells
TNF-α: tumor necrosis factor-α
IFN-γ: interferon-γ
PDI: polydispersity index
DCFH-DA: 2′,7′-dichlorofluorescin diacetate
UPK II: uroplakin II
TGI: tumor growth inhibition index
APCs: antigen-presenting cells
