# Supplementary material for: Enhanced efficacy of immune checkpoint inhibitors by folate-targeted multifunctional drug through synergistic therapy inducing ferroptosis and immunogenic cell death in bladder cancer
Source: Mater Today Bio. 2025 Feb 21;31:101584. doi: 10.1016/j.mtbio.2025.101584 (PMC11919378; doi:10.1016/j.mtbio.2025.101584)
Supplement: Multimedia component 1 [file mmc1.docx]

Table S1 Primary materials utilized in this experiment.

| Materials | Product No. | Company | Country |
| --- | --- | --- | --- |
| Doxorubicin hydrochloride | HY-15142 | MedChemExpress | USA |
| Indocyanine green | HY-D0711 | MedChemExpress | USA |
| Epidermal Growth Factor | HY-P7109 | MedChemExpress | USA |
| Ferrostatin-1 | HY-100579 | MedChemExpress | USA |
| Y-27632 | HY-10071 | MedChemExpress | USA |
| Methylene blue | 1428008 | Merck | USA |
| 1,3-diphenylisobenzofuran | 105481 | Merck | USA |
| Terephthalic Acid | 185361 | Merck | USA |
| Gentle Collagenase/Hyaluronidase | #07919 | STEMCELL | USA |
| TrypLE Express | 12605036 | Thermo Fisher | USA |
| RPMI 1640 medium | 11875085 | Thermo Fisher | USA |
| DMEM-F12 medium | 11320033 | Thermo Fisher | USA |
| Penicillin-Streptomycin Solution | C0222 | Beyotime | China |
| Penicillin-Streptomycin-Amphotericin B Solution | C0224 | Beyotime | China |
| Fetal bovine serum | ST30-3302 | PAN | Germany |
| Matrigel | 0827775 | ABW | China |
| Tislelizumab | / | Beigene | China |
| Cell counting kit-8 | C0037 | Beyotime | China |
| Calcein-AM/Propidium Iodide cell viability/cytotoxicity assay kit | C2015 | Beyotime | China |
| ROS detection kit | S0033 | Beyotime | China |
| GSH and GSSG assay kit | S0053 | Beyotime | China |
| Lipid Peroxidation MDA assay kit | S0131 | Beyotime | China |
| ATP assay kit | S0026 | Beyotime | China |
| HMGB1 ELISA kit | PH406 | Beyotime | China |
| Tumor Necrosis Factor-α (TNF-α) ELISA kit | PT518 | Beyotime | China |
| Interferon-γ (IFN-γ) ELISA kit | PI511 | Beyotime | China |
| FOLR1 Polyclonal antibody | 23355-1-AP | Proteintech | China |
| SLC7A11/xCT Polyclonal antibody | 26864-1-AP | Proteintech | China |
| GPX4 Monoclonal antibody | 67763-1-Ig | Proteintech | China |
| HMGB1 Polyclonal antibody | 10829-1-AP | Proteintech | China |
| Calreticulin Polyclonal antibody | 10292-1-AP | Proteintech | China |
| GAPDH Monoclonal antibody | 60004-1-Ig | Proteintech | China |
| CD4 Monoclonal antibody | 67786-1-Ig | Proteintech | China |
| CD8a Monoclonal antibody | 66868-1-Ig | Proteintech | China |
| CoraLite594 – conjugated Donkey Anti-Rabbit IgG(H+L) | SA00013-8 | Proteintech | China |
| CoraLite594 – conjugated Donkey Anti-Mouse IgG(H+L) | SA00013-7 | Proteintech | China |
| IRDye® 680RD Goat anti-Mouse IgG Secondary Antibody | 926-68070 | LI-COR | USA |
| IRDye® 800CW Goat anti-Rabbit IgG Secondary Antibody | 926-32211 | LI-COR | USA |
